# Building an EEG-fMRI Multi-Modal Brain Graph: A Concurrent EEG-fMRI Study

**DOI:** 10.3389/fnhum.2016.00476

**Published:** 2016-09-28

**Authors:** Qingbao Yu, Lei Wu, David A. Bridwell, Erik B. Erhardt, Yuhui Du, Hao He, Jiayu Chen, Peng Liu, Jing Sui, Godfrey Pearlson, Vince D. Calhoun

**Affiliations:** ^1^The Mind Research NetworkAlbuquerque, NM, USA; ^2^Department of Mathematics and Statistics, University of New MexicoAlbuquerque, NM, USA; ^3^School of Information and Communication Engineering, North University of ChinaTaiyuan, China; ^4^Department of Electrical and Computer Engineering, University of New MexicoAlbuquerque, NM, USA; ^5^Life Science Research Center, School of Life Sciences and Technology, Xidian UniversityShanxi, China; ^6^Brainnetome Center and National Laboratory of Pattern Recognition, Institute of Automation, Chinese Academy of SciencesBeijing, China; ^7^Olin Neuropsychiatry Research CenterHartford, CT, USA; ^8^Department of Psychiatry, Yale UniversityNew Haven, CT, USA; ^9^Department of Neurobiology, Yale UniversityNew Haven, CT, USA

**Keywords:** EEG-fMRI, dynamic, multi-modal, brain graph, ICA

## Abstract

The topological architecture of brain connectivity has been well-characterized by graph theory based analysis. However, previous studies have primarily built brain graphs based on a single modality of brain imaging data. Here we develop a framework to construct multi-modal brain graphs using concurrent EEG-fMRI data which are simultaneously collected during eyes open (EO) and eyes closed (EC) resting states. FMRI data are decomposed into independent components with associated time courses by group independent component analysis (ICA). EEG time series are segmented, and then spectral power time courses are computed and averaged within 5 frequency bands (delta; theta; alpha; beta; low gamma). EEG-fMRI brain graphs, with EEG electrodes and fMRI brain components serving as nodes, are built by computing correlations within and between fMRI ICA time courses and EEG spectral power time courses. Dynamic EEG-fMRI graphs are built using a sliding window method, versus static ones treating the entire time course as stationary. In global level, static graph measures and properties of dynamic graph measures are different across frequency bands and are mainly showing higher values in eyes closed than eyes open. Nodal level graph measures of a few brain components are also showing higher values during eyes closed in specific frequency bands. Overall, these findings incorporate fMRI spatial localization and EEG frequency information which could not be obtained by examining only one modality. This work provides a new approach to examine EEG-fMRI associations within a graph theoretic framework with potential application to many topics.

## Introduction

Graph theory-based analysis is a powerful technique to characterize the architecture of human brain networks (Avena-Koenigsberger et al., 2014; Pessoa, 2014). Graph metrics can quantitatively describe the topological properties of brain connectivity (Klimm et al., 2014). Previous studies that applied network science and graph theory based analysis to brain imaging data have reported “economical” small-world organization of connectivity, which reflects an economic balance between network cost and network efficiency (Bullmore and Sporns, 2012; Avena-Koenigsberger et al., 2014). The brain connectome shows modular and rich club organization with sets of hub regions that are crucial for efficient neuronal signaling and communication (van den Heuvel and Sporns, 2013b; Senden et al., 2014). Further studies show that the graph metrics and network structures of brain connectivity are altered in brain disorders (Collin et al., 2014; Crossley et al., 2014; Deco and Kringelbach, 2014; Hong et al., 2014; Korgaonkar et al., 2014; van den Heuvel and Fornito, 2014; Fornito and Bullmore, 2015a,b; Fornito et al., 2015; Gong and He, 2015; Wheeler et al., 2015). However, most of these studies have built the brain graphs with a single modality of brain imaging data. Additional insights of brain connectivity may thus be obtained by combining information from multiple modalities (Sui et al., 2012; Reid et al., 2016).

Different imaging techniques are sensitive to different aspects of brain dynamics. For example, functional magnetic resonance imaging (fMRI) measures the highly localized hemodynamic response throughout the brain, with a good spatial resolution (about 2–3 mm) but relatively poor temporal resolution. Electroencephalography (EEG) measures cortical electrical activity with a much higher temporal resolution, but its poor spatial resolution precludes precise anatomical identification of underlying neural sources. FMRI and EEG therefore represent complementary imaging signals, and combining concurrently collected data is a particularly useful way to examine brain dynamics over a broad range of spatial and temporal scales (Menon and Crottaz-Herbette, 2005; Herrmann and Debener, 2008; Eichele et al., 2009; Rosenkranz and Lemieux, 2010; Wu et al., 2010; Lei et al., 2011; Laufs, 2012; Bridwell et al., 2013; Mulert, 2013).

For coupling concurrent EEG-fMRI data, a popular approach is to analyze correlations between fMRI voxel time-series and EEG spectral power fluctuations (Valdes-Sosa et al., 2009; Rosa et al., 2010; Jorge et al., 2014). For example, it has been observed that low frequency EEG connectivity appears to best resemble fMRI connectivity (Deligianni et al., 2014). Brain connectivity between different regions detected by fMRI is associated with activity within different frequency bands of the EEG signal (Tagliazucchi et al., 2012; Chang et al., 2013; Liu et al., 2014). These findings provide electrophysiological signatures of functional brain connectivity identified in fMRI data (Mantini et al., 2007; Meir-Hasson et al., 2014). In this study, we make a further step to investigate topological organization of multi-modal EEG-fMRI brain connectivity.

Dynamic connectivity over time is an important feature in functional brain networks (Hutchison et al., 2013a,b; Li et al., 2013, 2014; Calhoun et al., 2014; Stephen et al., 2014; Yang et al., 2014), and dynamic connectivity patterns have been widely studied with fMRI (Allen et al., 2014; Rashid et al., 2014; Yu et al., 2015). Graph theory based analysis has been successfully implemented to assess dynamics during cognitive tasks or during rest (Doron et al., 2012; Bassett et al., 2013; Cocchi et al., 2013; Betzel et al., 2014; Cole et al., 2014; Dwyer et al., 2014; Hermundstad et al., 2014; Zalesky et al., 2014; Davison et al., 2015; Liang et al., 2016). A few recent studies even examined the relationships between dynamic fMRI connectivity and EEG signals (Tagliazucchi et al., 2012; Chang et al., 2013). However, the graph properties of dynamic brain networks with multi-modal nodes are largely unknown.

The aim of this study is to explore graph properties of concurrent EEG-fMRI multi-modal brain connectivity. Static and dynamic EEG-fMRI brain graphs are built using concurrently collected data from 25 healthy subjects during eyes open and eyes closed. Graph nodes are represented by EEG electrodes and fMRI components identified using group independent component analysis (ICA; Calhoun et al., 2008). Graph edges are represented by correlations between fMRI time courses and/or the EEG spectral power time courses of five frequency bands (delta, theta, alpha, beta, low gamma; for details, see the Methods Section below). The findings characterize changes within graphical properties of connectivity across different states (eyes open vs. eyes closed), while incorporating the high spatial resolution of fMRI (by estimating nodal graph measures of specific brain regions) and the high temporal resolution of EEG (i.e., we integrate fluctuations of fMRI brain regions with EEG frequencies within a graph-theoretic framework). In addition to the different spatial and temporal resolutions, different aspects of neural activity are also integrated between the two modalities, since EEG is sensitive to synchronous cortical synaptic potentials, and BOLD fMRI is sensitive to BOLD oxygenation changes that follow increased post-synaptic metabolism (see Bayram et al., 2011; Bridwell and Calhoun, 2014). This work develops a novel framework to build multi-modal brain graphs, demonstrating associations between EEG and fMRI within a graphical theoretical framework.

## Methods

### Participants

Twenty-five healthy subjects (age: 29 ± 8; 8 females) were recruited via advertisements at the University of New Mexico and by word-of-mouth. Each individual had normal or corrected to normal vision and hearing. Prior to inclusion in the study, participants were screened to ensure they were free from DSM-IV Axis I or Axis II psychopathology [assessed using the SCID (First et al., 1995)] and to ensure that there was no history of neurological disease. All participants provided informed written consent at the Mind Research Network, and were compensated for their participation. The experiment design and simultaneous acquisition details were described in our previous study (Wu et al., 2010).

### Experimental design

Simultaneous EEG-fMRI data were recorded while individuals rested first with their eyes closed (8.5 min), and then with their eyes open (8.5 min). Individuals were instructed to relax, lie still, and remain awake for the duration of each recording.

### EEG acquisition

EEG was recorded with a 32-channel BrainAmp MR-compatible system (Brainproducts, Munich, Germany) and a BrainCap electrode cap (Falk Minow Services, Herrsching-Breitbrunn, Germany). The Ag/AgCI electrodes were placed according to the international 10–20 system. Electrocardiogram (ECG) and eye movement (EOG) signals were recorded in separate channels, reducing the number of scalp electrodes to 30. The reference channel was placed at FCz. The impedance of each electrode was kept lower than 5 KΩ using conductive and abrasive electrode paste. The EEG signals were sampled at 5 KHz. To avoid temporal jitter, the EEG amplifier and fMRI were synchronized using an in-house device.

### fMRI acquisition

Functional MRI brain images were acquired with a Siemens Sonata (Siemens, Malvern PA) scanner at 1.5 T by means of a T2^*^-weighted echo planar imaging sequence with the following parameters: repeat time (TR) = 2 s, echo time (TE) = 39 ms, field of view = 224 mm, acquisition matrix = 64 × 64, flip angle = 80⋅, voxel size = 3.5 × 3.5 × 3 mm, gap = 1 mm, 27 slices, ascending acquisition. FMRI scans consisted of 256 volumes for each condition (eyes open and eyes close).

### EEG processing

EEG data were preprocessed in Matlab (http://www.mathworks.com) using custom and built-in functions and the EEGLAB toolbox (http://sccn.ucsd.edu/eeglab). The EPI gradient artifact was attenuated by calculating the average artifact template (across 2 s epochs) separately for each channel, and subtracting the template from the individual epochs within that channel. The EEG data were down-sampled to 1 kHz, band-pass filtered (0.01 to 50 Hz), and average referenced. Additional artifacts (e.g., BCG, eye movement, and residual EPI gradients) were attenuated by conducting a temporal ICA decomposition on the individual recordings (Srivastava et al., 2005). Thirty components were estimated using the extended Infomax algorithm implemented in EEGLAB (Bell and Sejnowski, 1995; Lee et al., 1999). Artifactual components were identified by visual inspection of the component time-course, topographic distribution, and frequency spectrum and removed from the back reconstructed time-course. Seventeen components were eliminated on average (min 11, max 23). In this work, ballistocardiac artifacts were corrected only by ICA. Previous studies showed that using ICA to attenuate the BCG artifacts in EEG data collected in a low magnetic field of 1.5 T is acceptable rather than in higher field scanners like 3 T or 7 T (Debener et al., 2008). We visually inspected the EEG waves and found the BCG artifacts were indeed largely removed. The experiment design, simultaneous acquisition details and data preprocessing were described in our previous studies (Wu et al., 2010; Bridwell et al., 2013). The data used in this work is the same as in these two studies.

The preprocessed EEG data were variance normalized, segmented into 2 s epochs (resulting in 256 epochs, i.e., an epoch that corresponds to each concurrently recorded fMRI volume) and converted to the frequency domain by the fast Fourier transform (FFT). The spectral power was averaged within 5 frequency bands (delta: 1–4 Hz; theta: 4–8 Hz; alpha: 8–13 Hz; beta: 13–30 Hz; low gamma: 30–35 Hz) for each epoch, resulting in 10 matrices (time [256] × electrodes [30]) of time series of spectral power for each subject (${SP}_{eyes\text{\_}open}^{delta}$; ${SP}_{eyes\text{\_}open}^{theta}$; ${SP}_{eyes\text{\_}open}^{alpha}$; ${SP}_{eyes\text{\_}open}^{beta}$; ${SP}_{eyes\text{\_}open}^{gamma}$; ${SP}_{eyes\text{\_}close}^{delta}$; ${SP}_{eyes\text{\_}close}^{theta}$; ${SP}_{eyes\text{\_}close}^{alpha}$; ${SP}_{eyes\text{\_}close}^{beta}$; ${SP}_{eyes\text{\_}close}^{gamma}$). The gamma band was restricted to lower frequencies (30–35 Hz) in order to avoid the pump and ventilation artifacts, which dominate above 40 Hz (Bridwell et al., 2013; Nierhaus et al., 2013). We noted that some other EEG literature define gamma as >40 Hz and call the band 30–35 Hz as high beta. However, in this study we name the band 30–35 Hz as low gamma.

### fMRI processing

FMRI data were preprocessed using SPM5 (http://www.fil.ion.ucl.ac.uk/spm/). Images were realigned using INRIalign (Freire and Mangin, 2001; Freire et al., 2002), spatially normalized to MNI space (Friston et al., 1995), subsampled to a voxel size of 3 × 3 × 3 mm, and smoothed with a Gaussian kernel (full width half maximum, FWHM, 5 × 5 × 5 mm).

One spatial group ICA (Calhoun et al., 2001, 2009; Rubinov et al., 2009; Calhoun and Adali, 2012; Du and Fan, 2013) was performed on the fMRI data of all subjects for the two conditions (i.e., both eyes open and eyes close) using the GIFT toolbox (http://mialab.mrn.org/software/gift). Subject-specific data reduction by principle component analysis (PCA) retained 120 (Erhardt et al., 2011) principal components (PCs) using a standard economy-size decomposition. Reduced data for all subjects were then decomposed into 100 (Kiviniemi et al., 2009; Smith et al., 2009; Abou-Elseoud et al., 2010; Abou Elseoud et al., 2011; Allen et al., 2011; Yu et al., 2015) aggregate components using the Infomax algorithm (Bell and Sejnowski, 1995). ICASSO analysis (http://research.ics.aalto.fi/ica/icasso; 10 iterations) indicated that the components were stable (see Figure S1). Single subject independent components (ICs) and associated time courses (TCs) were back-reconstructed (Calhoun et al., 2001; Erhardt et al., 2011). Fifty-four ICs were characterized as intrinsic connectivity networks (ICNs), as opposed to physiological, movement related, or imaging artifacts (ARTs; Allen et al., 2014; Yu et al., 2015). The components were evaluated based on expectations that ICNs should exhibit peak activations in gray matter, low spatial overlap with known vascular, ventricle, motion, and susceptibility artifacts and should have TCs dominated by low-frequency fluctuations (<0.1 Hz; Cordes et al., 2000). Following Allen et al. (2014), TCs of the 54 ICs underwent additional post-processing including (1) detrending linear, quadratic, and cubic trends, (2) multiple regression of the 6 realignment parameters and their temporal derivatives, (3) removal of detected outliers (despiking), and (4) band-pass filtering with frequency band [0.01–0.10 Hz]. Finally, ICA time course matrices [time: 256 × ICNs: 54, FT (256 × 54)] were derived for each condition (eyes open and eyes close) for each subject.

### Building EEG-fMRI brain graphs

A correlation matrix R was constructed with elements (r_ij_) representing Pearson correlation coefficients computed using the 30 EEG electrodes' spectral time-courses and the 54 fMRI ICs' time-courses. This process was repeated for the five EEG frequency bands and the two conditions (EO and EC). When computing the correlation between EEG and fMRI signals, following previous studies (Goldman et al., 2002; Laufs et al., 2003a; Moosmann et al., 2003), EEG power time courses were convolved with a canonical hemodynamic response function (HRF) to account for the delayed hemodynamic response.

Consequently, undirected static connectivity EEG-fMRI graphs were built from each of the *N* × *N* (*N* = 84 in this study, including 30 EEG electrodes and 54 fMRI brain components) correlation matrices R. In order to preserve the information for both positive and negative correlations, weighted positive (*W*^+^) and negative (*W*^−^) connection graphs were built based on *R*. In positive connection graphs, negative correlations in *R* were replaced by 0 and positive correlation values were maintained. In negative connection graphs, positive correlations were replaced by 0 and absolute values of negative correlations in R were maintained.

(1) $$w_{ij}^{+}=\{\begin{array}{c} r_{ij}\;if\;r_{ij}\;>\;0\\ 0\;if\;r_{ij}\;\leq \;0 \end{array}$$

(2) $$w_{ij}^{-}=\{\begin{array}{c} \left|r_{ij}\right|\;if\;r_{ij}\;<\;0\\ 0\;if\;r_{ij}\;\geq \;0 \end{array}$$

**Figure 1:**
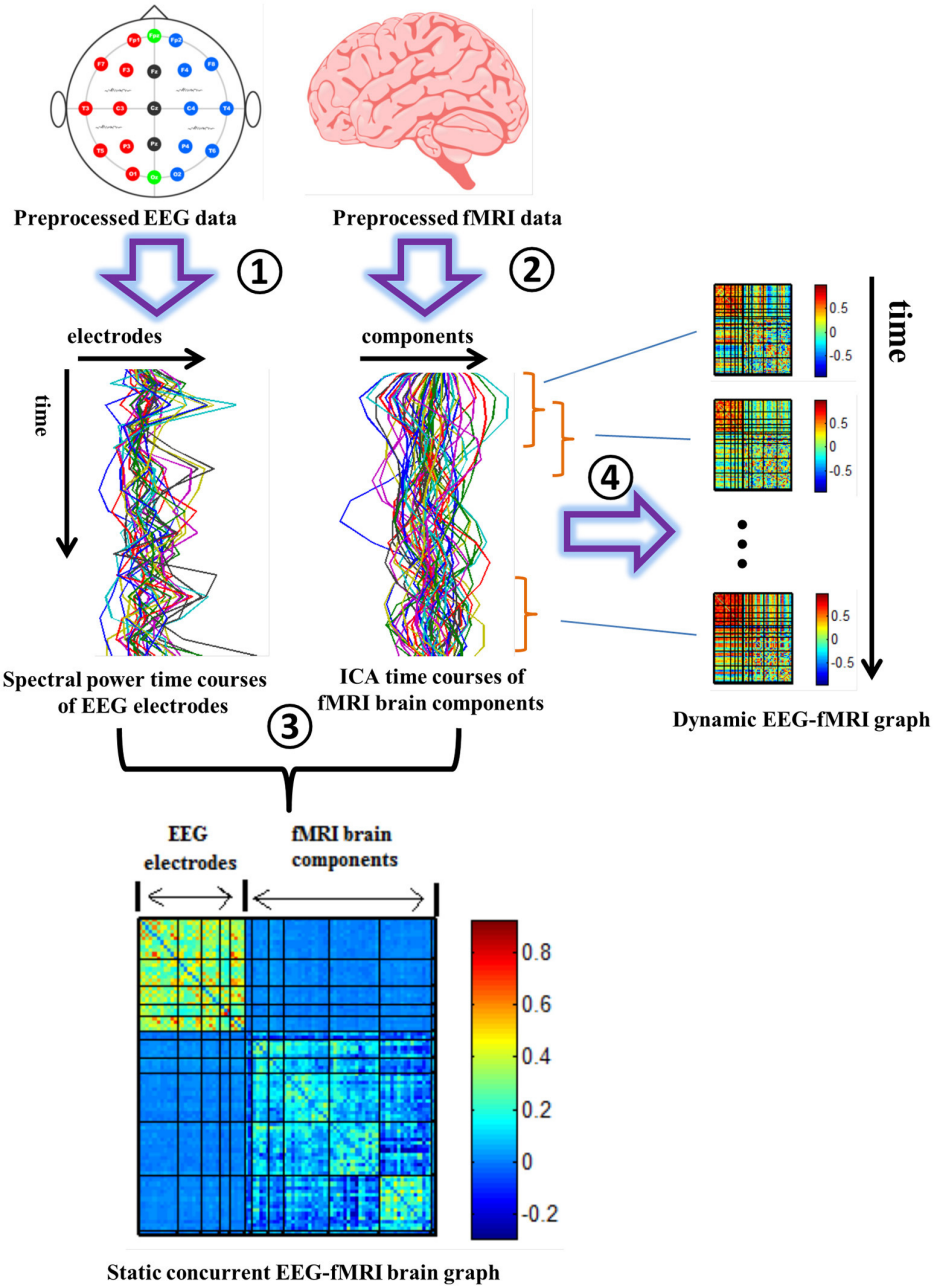
**Pipeline for building concurrent EEG-fMRI multi-modal brain graphs**. ① Segment EEG signal into 2 s time windows, and compute the average spectral power within a selected frequency window. ② Perform group ICA on fMRI data. ③ Compute the correlation coefficient within and across the EEG spectral power's and fMRI ICA's full time courses, generating one EEG-fMRI static connectivity matrix for each frequency band. ④ Segment EEG spectral power and fMRI ICA time courses into time windows, then compute the correlation between each pair of time windowed time courses to get dynamic EEG-fMRI brain graphs. Both positive and negative correlations in the correlation matrix (R) are shown in this figure. These steps are repeated for each of the 5 frequency bands and during eyes open and eyes closed conditions.

Dynamic EEG-fMRI graph analysis was performed by calculating correlation matrices along successive sliding windows of the matrix EF (256 × 84; width, L = 20 TRs, in steps of 1 TR; Allen et al., 2014; Yu et al., 2015). As with the static analysis, the first 30 columns correspond to EEG electrodes and the following 54 columns correspond to fMRI ICs. Two hundred thirty-seven EEG-fMRI correlation matrices were computed for 237 (237 = 256 − 20 + 1) windows. Positive and negative connection graphs were analyzed separately, as in the static analysis. See Figure 1 for the framework of building static and dynamic concurrent EEG-fMRI multi-modal brain graphs.

Here we use a window width of 20 TRs (40 s) based on a previous study indicating that cognitive states may be correctly identified with as little as 30–60 s of data (Shirer et al., 2012). A recent study has shown that non-stationary fluctuations in functional connectivity can in theory be detected with window length of 40 s (Zalesky and Breakspear, 2015). Also, others have demonstrated that changes of brain connectivity are not particularly sensitive to the specific time-window length (in the range of 10–20 2 s TRs, 20–40 s; Li et al., 2014). Previous works of our group and others consistently show that shorter time windows result in a lower number of statistically significant correlations in brain connectivity and greater variability of correlation values (Chang and Glover, 2010; Hutchison et al., 2013b; Allen et al., 2014; Yu et al., 2015), whereas a sliding window size of about 22 TRs (44 s) provides a good trade-off between the ability to resolve dynamics and the quality of connectivity estimation (Allen et al., 2014; Yu et al., 2015).

Connectivity strength (CS), clustering coefficient (CC), and global efficiency (GE) are three basic and important graph metrics which measure the functional segregation and integration of brain networks (Rubinov and Sporns, 2010). To quantitatively assess the topological properties of the brain connectivity, both global level and nodal level of these three graph metrics were derived for static and dynamic graphs in this study using the brain connectivity toolbox (http://www.brain-connectivity-toolbox.net/). For mathematical definitions and equations for computing the graph measures see the below Section Equations for Computing Graph Measures. More details are available in (Rubinov and Sporns, 2010)]. The variances of 237 dynamic graph metrics and the amplitude of low frequency (0–0.025 Hz) fluctuations of the time series of dynamic graph measures were computed in each subject. For statistical analysis, 5 (frequency band) × 2 (eyes condition) compound symmetry repeated measure analysis of variance (ANOVA) and paired *t*-tests were performed on static and dynamic measures.

### Equations for computing graph measures

We denote *G* as the set of all nodes in the weighted graph *W*, and *N* (*N* = 84 in this study) is the number of nodes. Connectivity strength of node i is defined as below:

(3) $$\begin{array}{l} {CS}_{i}=\sum_{j\epsilon G}w_{ij} \end{array}$$

The connectivity strength of whole graph (global level) is the average of the connectivity strength of all the nodes in the graph:

(4) $$\begin{array}{l} {CS}_{net}=\frac{1}{N}\sum_{i\epsilon G}{CS}_{i} \end{array}$$

Nodal level Clustering coefficient is computed using the below equation:

(5) $$\begin{array}{l} {CC}_{i}=\frac{1}{{CS}_{i}\left({CS}_{i}-1\right)}\sum_{j,k\epsilon G}{\left(w_{ij}w_{ik}w_{jk}\right)}^{1∕3} \end{array}$$

The clustering coefficient of whole graph is the average of the clustering coefficient of all the nodes in the graph:

(6) $$\begin{array}{l} {CC}_{net}=\frac{1}{N}\sum_{i\epsilon G}{CC}_{i} \end{array}$$

Global efficiency of node *i* is defined as:

(7) $$\begin{array}{l} {GE}_{i}=\frac{\sum_{j\epsilon N,j\;\neq \;i}{\left(d_{ij}\right)}^{-1}}{N-1} \end{array}$$

In which

(8) $$\begin{array}{l} d_{ij}=\sum_{a_{uv}\epsilon g_{i\overset{w}{↔}j}}f\left(w_{uv}\right) \end{array}$$

Where *f* is a map (here is an inverse) from weight to length and $g_{i}\overset{w}{↔}\text{j}$ is the shortest weighted path between *i* and *j*. Global efficiency of the whole graph is the average of the global efficiency of all the nodes in the graph.

### Detecting connectivity states

Recent fMRI studies showed that fluctuations of time-varying functional brain connectivity gives rise to discrete highly-organized patterns that may emerge or dissolve over time, which are called connectivity states (Cribben et al., 2012; Allen et al., 2014; Yang et al., 2014; Yu et al., 2015). Here we performed the method developed in one of our previous studies (Yu et al., 2015) to detect connectivity states of the dynamic EEG-fMRI graphs in each individual. Firstly, nodal level connectivity strength of each time-varying EEG-fMRI graph was computed. To estimate how the EEG-fMRI network patterns of different time-windows were associated to each other, a new correlation matrix CCS (237 × 237; 237 is the number of time windows) was then computed based on correlations of the nodal connectivity strength between each pair of time windows across 84 nodes. Modular community structure is one of the most ubiquitous properties of complex networks (Newman, 2006; Bullmore and Sporns, 2009). Modularity is a function that measures the quality of a division of nodes into groups or communities, and modules of the matrix CCS may correspond to sets of time windows with similar brain connectivity patterns. Thus, the modular organization of CCS was analyzed with the modularity algorithm of Newman (2006) implemented in the brain connectivity toolbox. The number of modules of CCS is the number of connectivity states for the dynamic EEG-fMRI graph. Finally, the EEG-fMRI brain graphs from different time windows that categorized to the same module were averaged to get the graph of that connectivity state. More details of this approach were introduced in (Yu et al., 2015).

## Results

### Spatial maps of fMRI brain components

**Figure 2:**
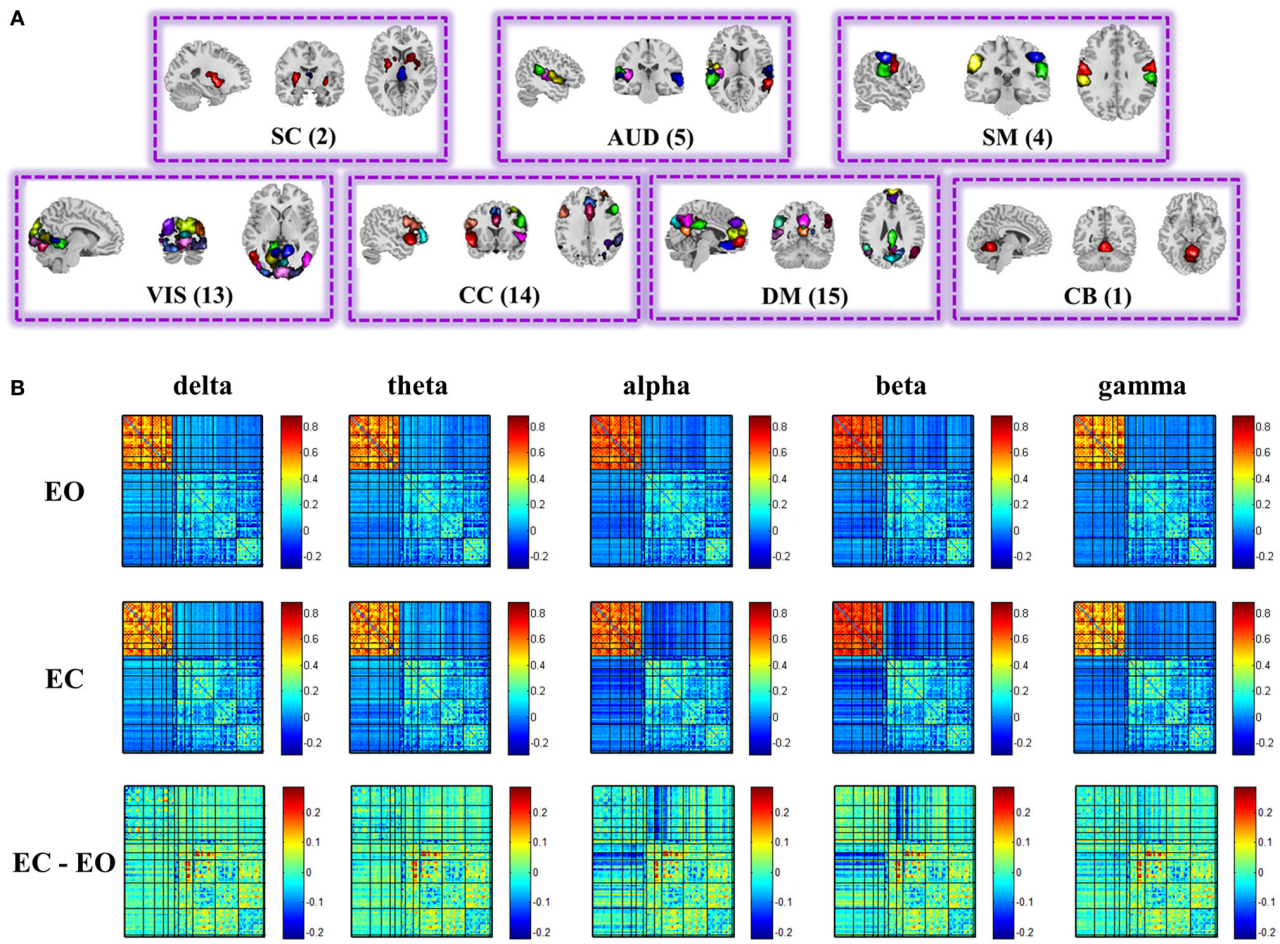
**Spatial maps of the 54 ICs (A), and structure of the group mean static EEG-fMRI brain graphs in the five frequency bands for eyes open, eyes closed, and difference between eyes conditions (B)**. Both positive and negative correlations of the correlation matrix are shown in this figure. See Figure S6 for the nodes organization. (EO, eyes open; EC, eyes closed; SC, sub-cortical; AUD, auditory; SM, somatomotor; VIS, visual; CC, cognitive control; DM, default-mode; CB, cerebellar).

Figure 2A displays the spatial maps of the 54 ICNs identified with group ICA. Based on their anatomical and presumed functional properties, 54 ICNs are arranged into groups of subcortical (SC), auditory (AUD), somato-motor (SM), visual (VIS), cognitive control, default-mode (DM), and cerebellar (CB) components. ICNs are similar to those observed in previous high model order ICA decompositions (Abou-Elseoud et al., 2010; Allen et al., 2014; Yu et al., 2015), and a subset have been associated with cognitive functions in meta-analytic studies (Rottschy et al., 2012; Balsters et al., 2014; Kohn et al., 2014; Amft et al., 2015).

### Static EEG-fMRI graph

Figure 2B displays the structure of stationary connectivity between graph nodes (ICNs and EEG channels), computed over the entire fMRI time courses and EEG spectra power time courses for the five frequency bands (delta, theta, alpha, beta, low gamma) and averaged over 25 subjects in each condition (eyes open and eyes closed). Patterns of connectivity within fMRI ICNs are consistent with prior literature, showing modular organization within sensory systems and default mode regions, as well as anticorrelation between these regions (Fox et al., 2005; Shirer et al., 2012; Allen et al., 2014; Yu et al., 2015).

For the global level graph metrics of positive connection networks, a five (frequency band: delta, theta, alpha, beta, low gamma) × two (eyes condition: open, closed) compound symmetry repeated measures ANOVA shows that the main effect of frequency band is significant (*P* < 0.001) for all three metrics, and the main effect of eyes condition is significant (*P* < 0.01) on clustering coefficient only. *Post-hoc* paired *t*-tests reveal significantly (*P* < 0.01) higher clustering coefficient during eyes closed than eyes open in beta band.

For the global level graph metrics of negative connection networks, a five (frequency band: delta, theta, alpha, beta, low gamma) × two (eyes condition: open, closed) compound symmetry repeated measures ANOVA shows that the main effects of frequency band and eyes condition are significant (*P* < 0.05) for all three metrics (connectivity strength, clustering coefficient, global efficiency). *Post-hoc* paired *t*-tests reveal significantly (*P* < 0.01) higher connectivity strength during eyes closed than eyes open in alpha and beta bands, and significantly higher global efficiency during eyes closed in the beta band.

Figures S2, S3 show the group means of the graph metrics in the eyes closed and eyes open conditions computed within the delta, theta, alpha, beta and low gamma frequency bands of positive and negative connection graphs, respectively.

For nodal level graph metrics of positive connection graphs, the main effect of eyes condition is significant (FDR correction, *q* < 0.001) on all three graph metrics for 3 brain components which belong to somatomotor, visual, and auditory components, respectively. Graph measures are higher during eyes closed than during eyes open. For the spatial maps of the 3 ICNs see Figure S4.

For nodal level graph metrics of negative connection graphs, the main effect of eyes condition is significant (FDR correction, *q* < 0.001) on all three graph metrics for only one visual brain components. Graph measures are higher during eyes closed than during eyes open. For the spatial maps of that ICN see Figure S5.

**Figure 3:**
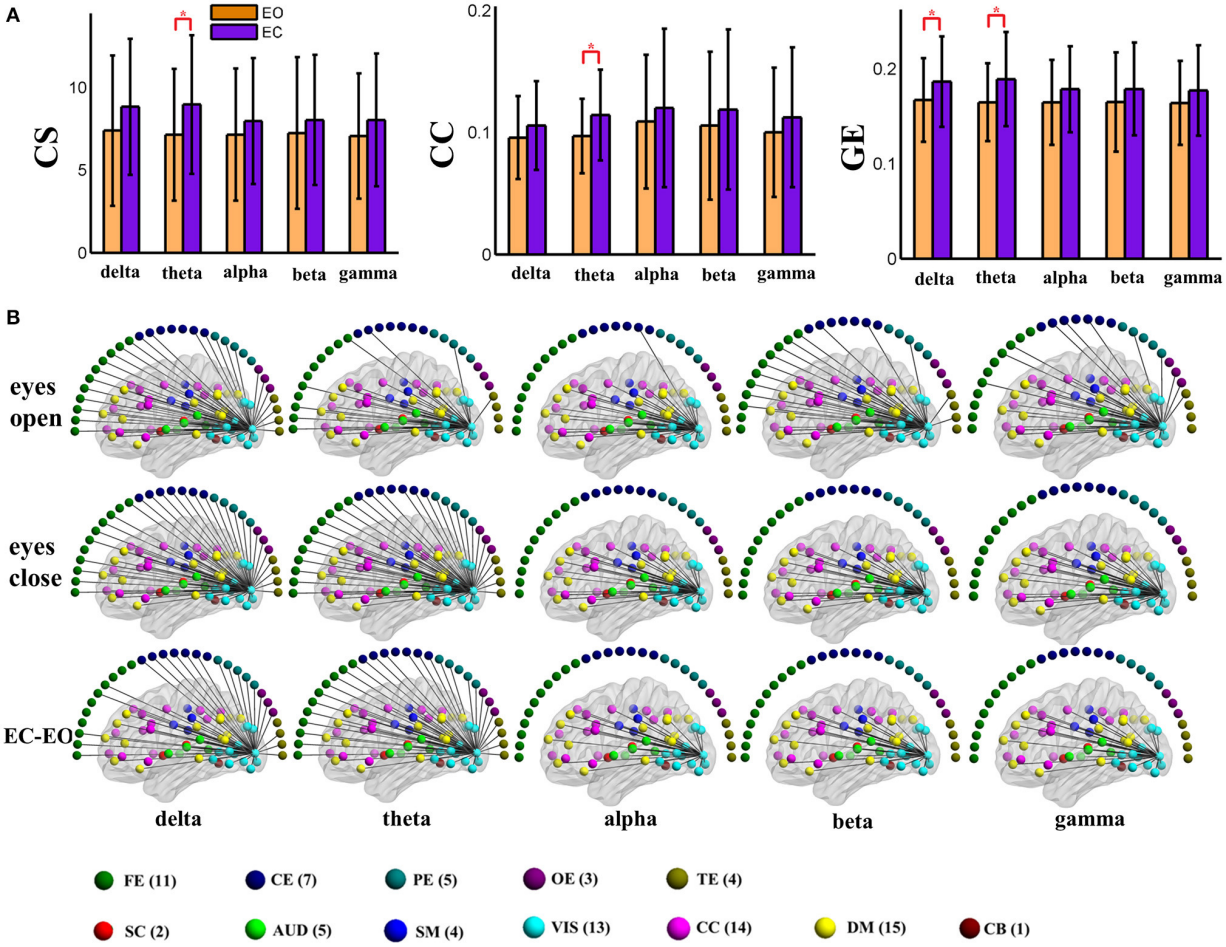
**(A)** Graph metrics of a visual component node in the static positive connection graphs. Connectivity strength is indicated on the left, the clustering coefficient is indicated in the middle, and global efficiency is indicated on the right. The bar color indicates the eyes (open vs. closed) condition and the bar height indicates the mean value of the measurement for the 25 subjects. Error bars correspond to standard deviations. The main effect of the eyes condition is significant for CS and GE (FDR correction, *q* < 0.001), with higher values during eyes closed than during eyes open. (“*” indicates *P* < 0.05 for *post-hoc* paired *t*-test). **(B)** Patterns of connections from one visual component node to other nodes are indicated for the different frequency bands and conditions. The graphs are plotted using the same threshold across all 5 frequency bands in eyes open, eyes closed or the difference between the two conditions. Only positive connections are shown in the graphs of difference between conditions. Color dots inside the brain map indicate fMRI brain components. Color dots outside the brain map indicate EEG electrodes. (CS, connectivity strength; CC, clustering coefficient; GE, global efficiency; EC, eyes closed; EO, eyes open; FE, frontal electrodes; CE, central electrodes; PE, parietal electrodes; OE, occipital electrodes; TE, temporal electrodes).

**Figure 4:**
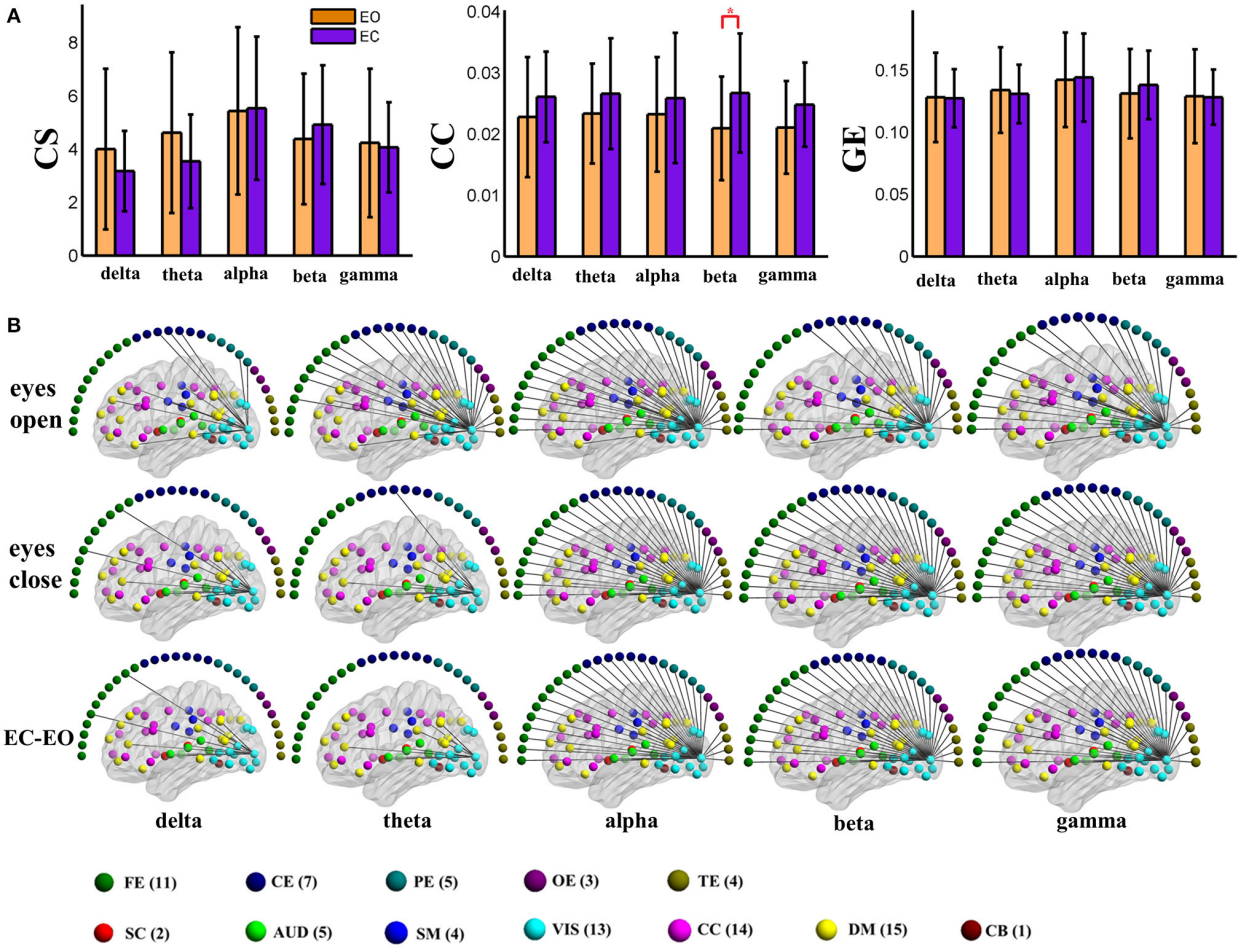
**(A)** Graph metrics of a visual component node in the static negative connection graph. Connectivity strength is indicated on the left, the clustering coefficient is indicated in the middle, and global efficiency is indicated on the right. The bar color indicates the eyes (open vs. closed) condition and the bar height indicates the mean value of the measurement for the 25 subjects. Error bars correspond to standard deviations. The main effect of the eyes condition is significant for CC (FDR correction, *q* < 0.001), with higher values during eyes closed than during eyes open. (“*” indicates *P* < 0.05 for *post-hoc* paired *t*-test). **(B)** Patterns of connections from one visual component node to other nodes are indicated for the different frequency bands and conditions. The graphs are plotted using the same threshold across all 5 frequency bands in eyes open, eyes closed or the difference between the two conditions. Only positive connections are shown in the graphs of difference between conditions. Color dots inside the brain map indicate fMRI brain components. Color dots outside the brain map indicate EEG electrodes. (CS, connectivity strength; CC, clustering coefficient; GE, global efficiency; EC, eyes closed; EO, eyes open; FE, frontal electrodes; CE, central electrodes; PE, parietal electrodes; OE, occipital electrodes; TE, temporal electrodes).

To visually display the difference between eyes conditions of the brain network, as an example, we show the values of the nodal graph measures and the pattern of the connections from a visual component node to all of the other graph nodes in the five frequency bands during eyes open and eyes closed conditions in positive and negative connection graphs in Figures 3, 4, respectively.

### Dynamic EEG-fMRI graph

Figures S7–S16 display the global graph metrics including connectivity strength (CS), clustering coefficient (CC), and global efficiency (GE) of both positive connection and negative connection time varying EEG-fMRI brain connectivity (237 time windows) in the five frequency bands for all 25 subjects in eyes open and eyes closed conditions. These figures indicate the changes in graph metrics over time. Figures S17, S18 show positive connection and negative connection time varying global level connectivity strength of an example subject, respectively. As demonstrated from the CS time series (Figures S17, S18, A1, A2), the CS is highly non-stationary [Kwiatkowski-Phillips-Schmidt-Shin (KPSS) tests, *P* < 0.01]. Fourier analysis of the time series (Figures S17, S18, B1, B2) shows that low-frequency CS oscillations peak between 0.001 and 0.02 Hz.

**Figure 5:**
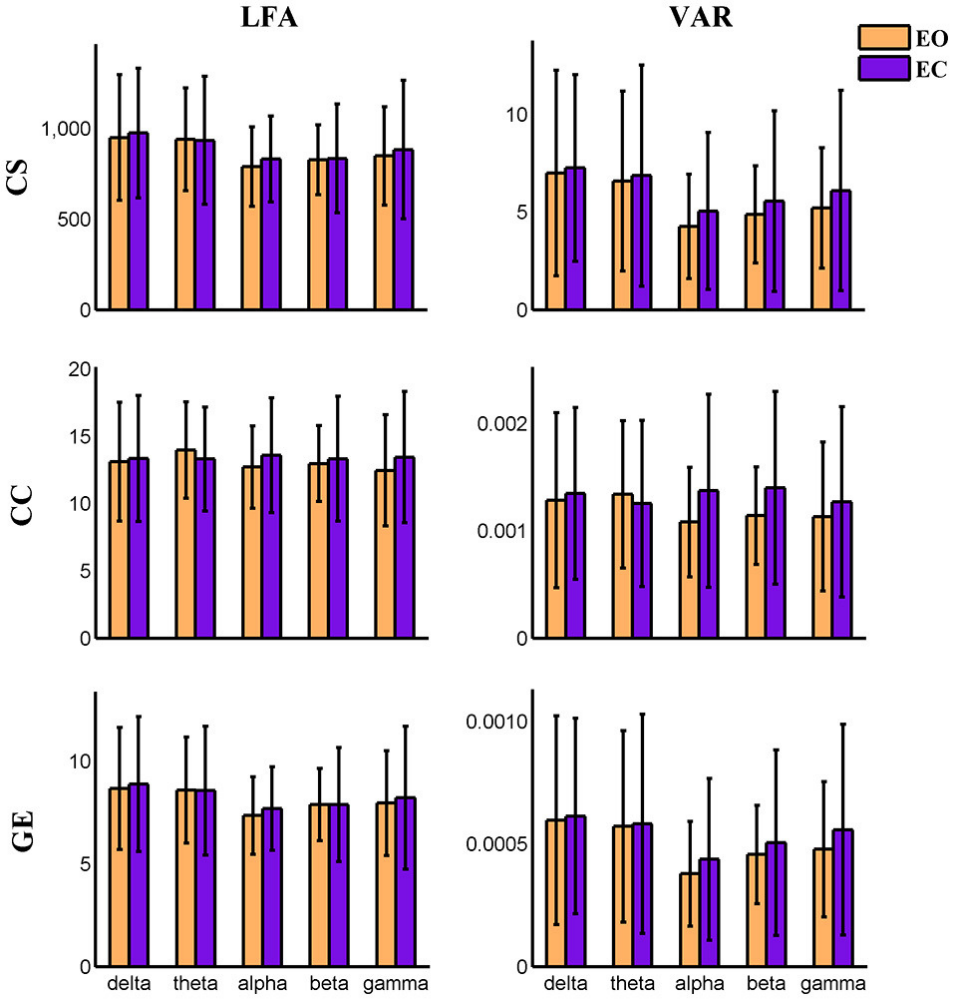
**Amplitude of low frequency (LFA) and variance (VAR) of the time series of three global level graph measures in time-varying positive connection graphs**. The bar color indicates the eyes condition and the bar height indicates the mean value of VAR or LFA for the 25 subjects. Error bars correspond to standard deviations. The main effect of eyes condition is not significant for LFA and VAR of dynamic graph measures. The main effect of frequency band is significant (*P* < 0.01) on VAR and LFA of CS and GE. (CS, connectivity strength; CC, clustering coefficient; GE, global efficiency).

**Figure 6:**
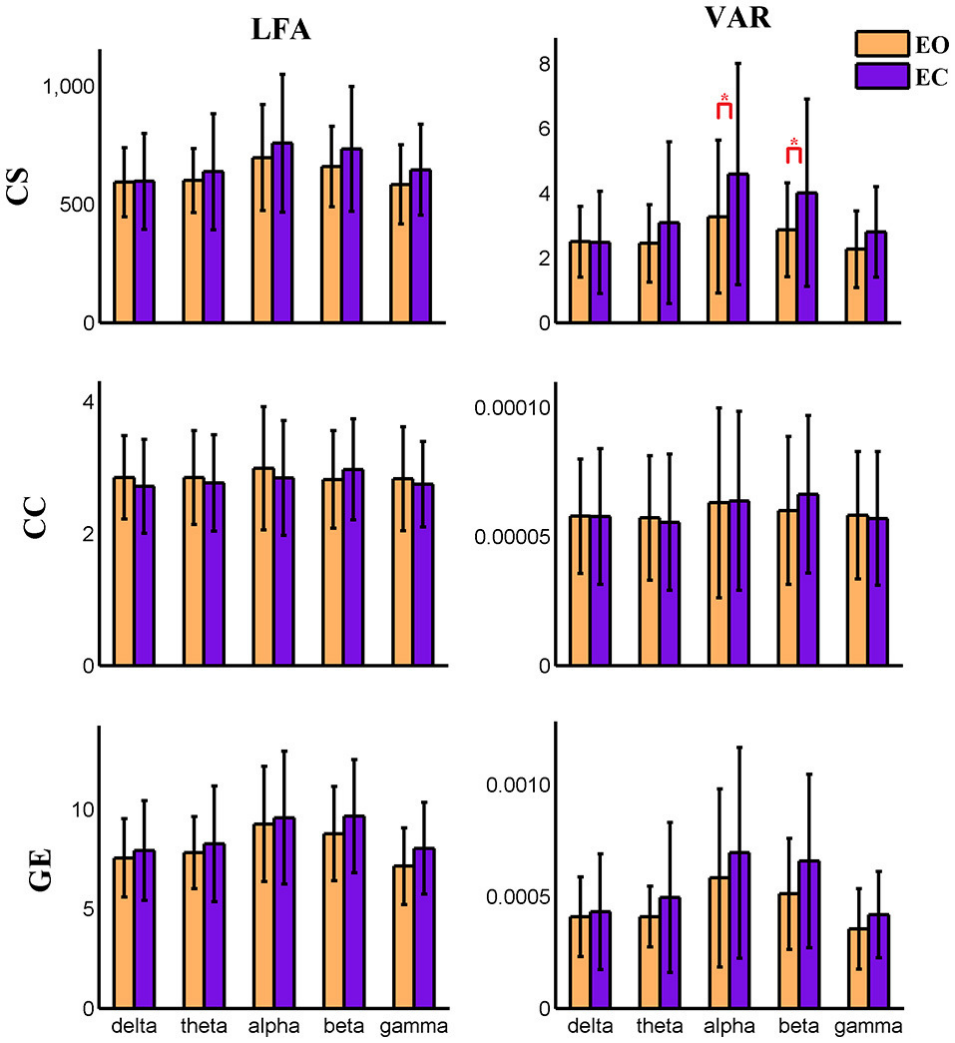
**Amplitude of low frequency (LFA) and variance (VAR) of dynamic global graph metrics in time-varying negative connection graphs**. The bar color indicates the eyes condition and the bar height indicates the mean value of the measurement for the 25 subjects. Error bars correspond to standard deviations. The main effect of eyes condition is significant (*P* < 0.05) for the LFA of CS and the VAR of CS and GE. The main effect of frequency band is significant (*P* < 0.001) on VAR and LFA of CS and GE. (“*” indicates *P* < 0.05 for paired *t*-test; CS, connectivity strength; CC, clustering coefficient; GE, global efficiency).

Variance (VAR) and amplitude of low frequency (LFA) [0–0.025 Hz] oscillations of the time varying global level graph metrics are computed. For time-varying positive connection graphs, five (frequency band: delta, theta, alpha, beta, low gamma) × two (eyes condition: open, close) compound symmetry repeated measure ANOVA shows that the main effect of eyes condition is not significant on VAR and LFA of all dynamic graph measures. The main effect of frequency band is significant (*P* < 0.01) on VAR and LFA of CS and GE (see Figure 5), indicating that the graph metrics computed using delta and theta EEG frequencies demonstrate low frequency patterns of time varying connectivity. For time-varying negative connection graphs, the main effect of eyes condition is significant (*P* < 0.01) on VAR of CS and GE, and on LFA of CS (*P* < 0.05). The main effect of frequency band is significant (*P* < 0.001) on VAR and LFA of CS and GE (see Figure 6). In general, we found greater variance (VAR) and LFA in CS and GE in eyes closed alpha compared to eyes open alpha.

**Figure 7:**
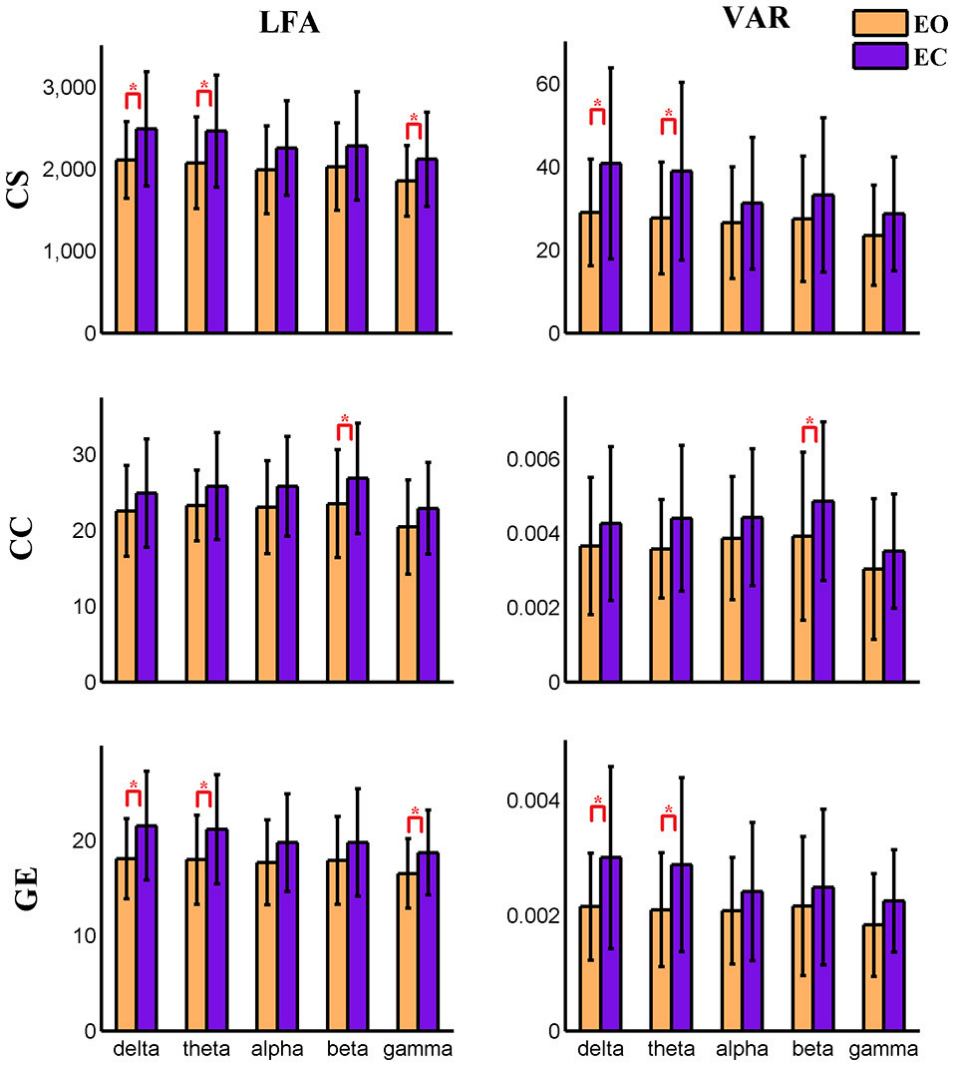
**Amplitude of low frequency (LFA) and variance (VAR) of the time series of three graph measures of a visual component node in time-varying positive connection graphs**. The bar color indicates the eyes condition and the bar height indicates the mean value of VAR or LFA for the 25 subjects. Error bars correspond to standard deviations. The main effect of eyes condition is significant (FDR correction, *q* < 0.001) for LFA and VAR of CS and GE. Values of VAR and LFA are higher in eyes closed than in eyes open and paired *t*-tests show significant difference between eyes open and eyes closed for CS and GE mainly in delta and theta bands. The main effect of frequency band is not significant for any measure. (“*” indicates *P* < 0.05 for paired *t*-test; CS, connectivity strength; CC, clustering coefficient; GE, global efficiency).

**Figure 8:**
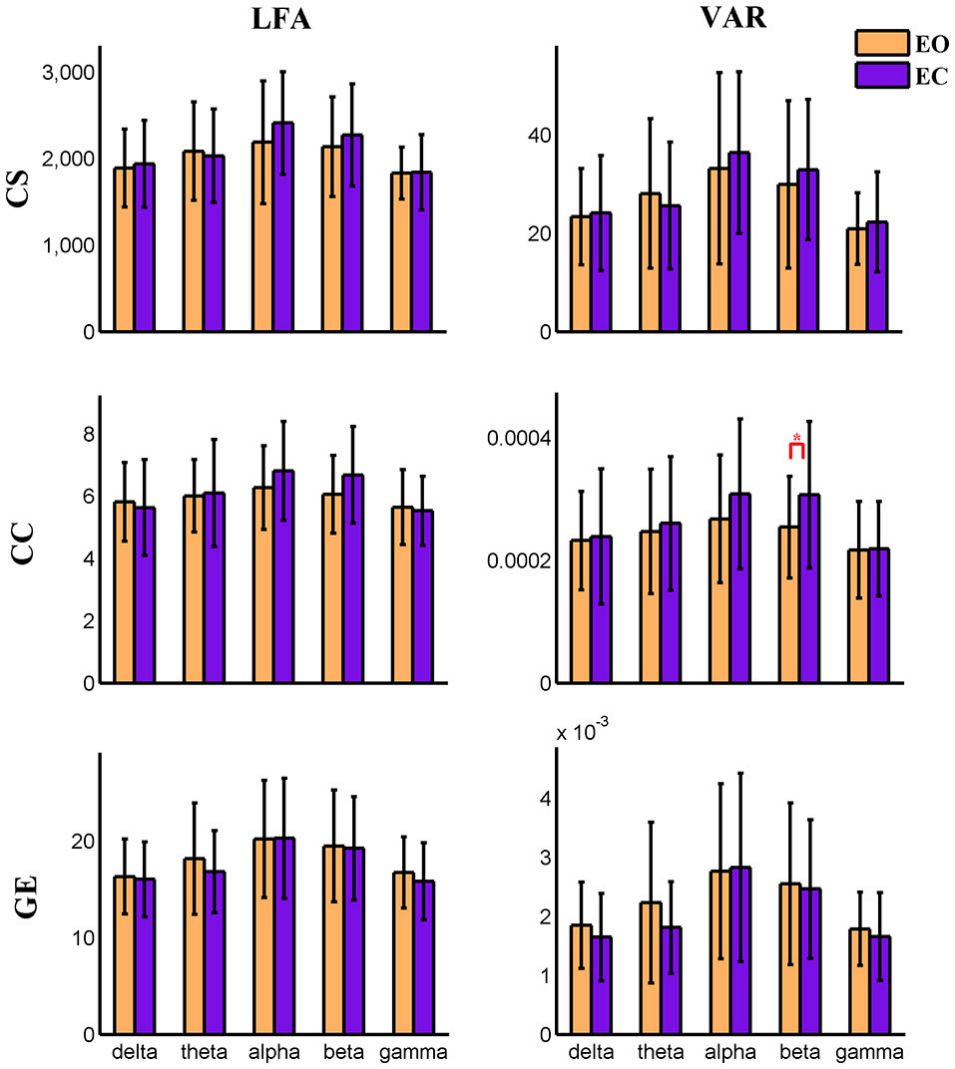
**Amplitude of low frequency (LFA) and variance (VAR) of dynamic graph metrics of a visual component node in time-varying negative connection graphs**. The bar color indicates the eyes condition and the bar height indicates the mean value of the measurement for the 25 subjects. Error bars correspond to standard deviations. The main effect of eyes condition is not significant for any measure. The main effect of frequency band is significant (FDR correction, *q* < 0.001) for all measures. (“*” indicates *P* < 0.05 for paired *t*-test; CS, connectivity strength; CC, clustering coefficient; GE, global efficiency).

**Figure 9:**
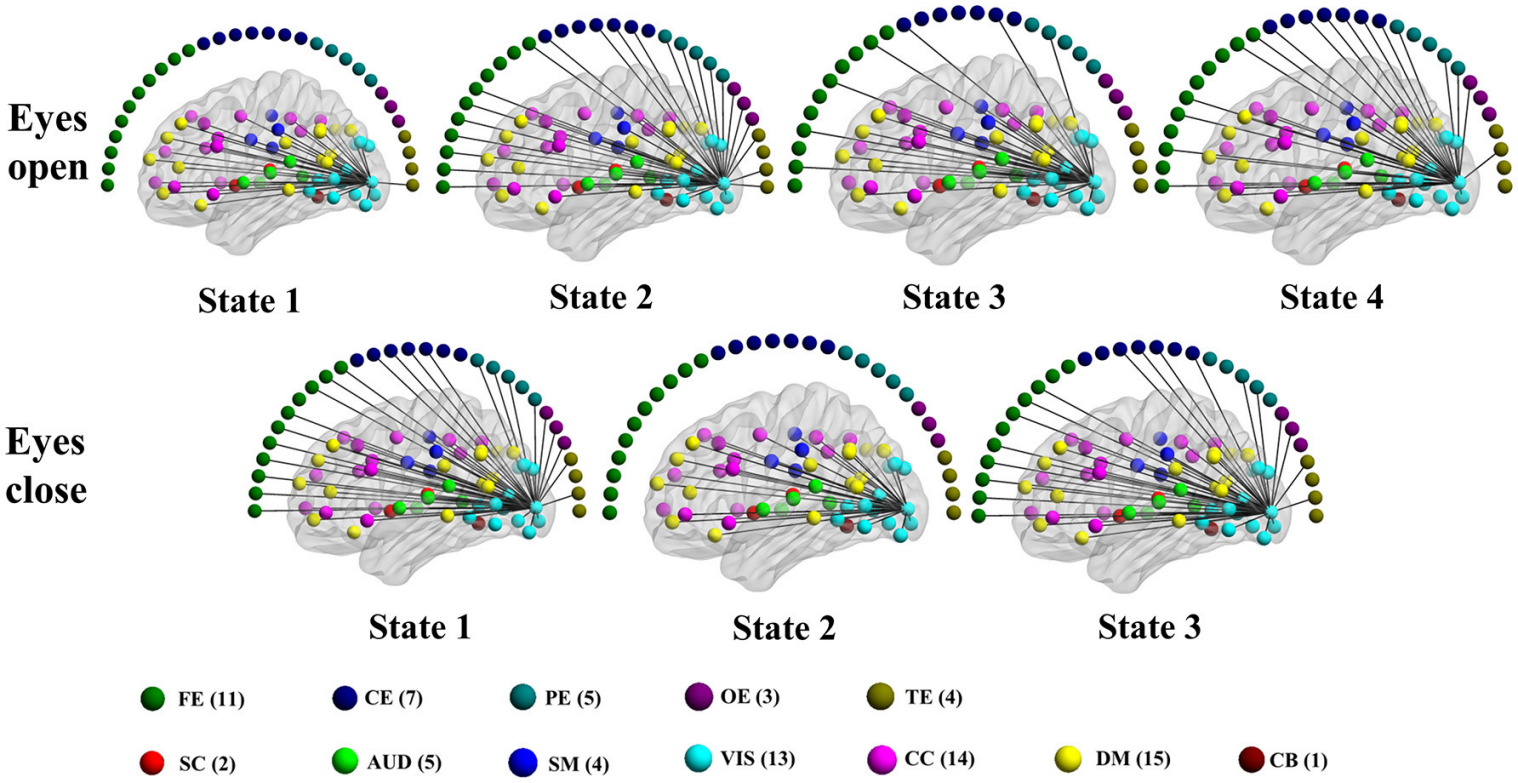
**Patterns of connections from a visual component node to all other graph nodes for the connectivity states identified in an example subject in the alpha band for time-varying positive connection graphs**. Color dots inside the brain map indicate the approximate centroid location of fMRI brain components. Color dots outside the brain map indicate EEG electrodes. (FE, frontal electrodes; CE, central electrodes; PE, parietal electrodes; OE, occipital electrodes; TE, temporal electrodes).

**Figure 10:**
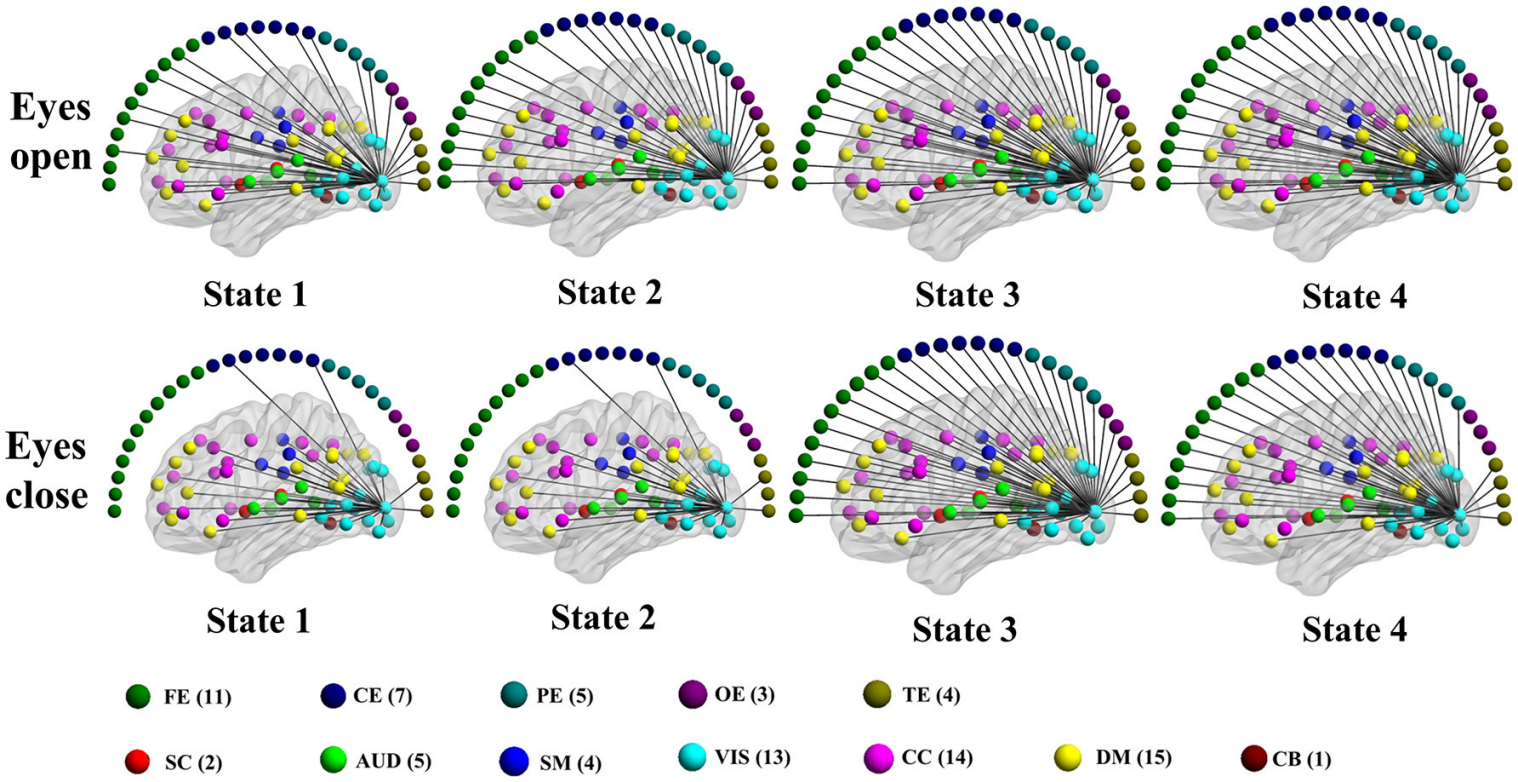
**Patterns of connections from a visual component node to all other graph nodes for the connectivity states identified in an example subject in the alpha band for time-varying negative connection graphs**. Color dots inside the brain map indicate the approximate centroid location of fMRI brain components. Color dots outside the brain map indicate EEG electrodes. (FE, frontal electrodes; CE, central electrodes; PE, parietal electrodes; OE, occipital electrodes; TE, temporal electrodes).

For nodal level dynamic graph metrics of positive connection networks, the main effect of eyes condition on the VAR and LFA of all three dynamic measures is significant (FDR correction, *q* < 0.001) at two cognitive control brain components. For the spatial map of these two components see Figure S19. One visual component shows significant (FDR *q* < 0.001) main effect of eyes condition on VAR and LFA of two metrics (CS and GE). See Figure S20 for its spatial map. The same component is shown in (Figures 3, 4, **9**, **10**). To demonstrate the dynamic properties of nodal level graph metrics, we show VAR and LFA of the visual component as an example in Figures 7, 8.

### Connectivity states

Consistent with previous dynamic fMRI connectivity studies (Allen et al., 2014; Damaraju et al., 2014; Rashid et al., 2014; Yang et al., 2014), some connectivity patterns of the dynamic EEG-fMRI graphs reoccur over time. Connectivity states are detected using the method developed in our previous study (Yu et al., 2015). Specifically, 2–6 states are detected during each eyes condition in each subject for both positive and negative. See Tables S1, S2 for the details about how many connectivity states are detected in each subject. For a visual view of the structure of different connectivity states in an example subject, see Figures S21–S24, and Figures 9, 10.

## Discussion

In the present study, concurrent EEG-fMRI resting state data collected during eyes open and eyes closed conditions are used to build multi-modal brain graphs. FMRI data are decomposed with group ICA into ICNs and corresponding time courses. EEG signals are segmented into 2 s-epochs and the spectral power is computed and averaged within five frequency bands (delta, theta, alpha, beta, and low gamma) for each segment. EEG-fMRI brain graphs are built by computing the correlations between and among fMRI ICA time courses and EEG spectral power time courses. Connectivity strength, local efficiency, and global efficiency are calculated for both static graphs, which are estimated using the full length of time courses, and dynamic graphs, which are estimated using a sliding window method. Five (frequency band: delta, theta, alpha, beta, low gamma) × two (eyes condition: open, close) compound symmetry repeated measure ANOVA and paired *t*-tests are performed to identify significant differences across frequencies and eyes conditions. For static graphs, in positive connection networks, graph metrics are higher during the eyes closed condition compared to eyes open mainly in delta and theta bands (Figure 3). In negative connection networks, graph metrics are higher during eyes closed compared to eyes open primarily in alpha and beta bands (Figure 4, Figure S3). For time varying graphs, in positive connection networks, the LFA and VAR of dynamic graph measures (in nodal level of specific nodes) are higher in eyes closed than eyes open mainly in delta and theta bands (Figure 7). In both positive and negative connection networks, generally, we found greater variance (VAR) and LFA in CS and GE in eyes closed alpha compared to eyes open alpha (Figures 5, 6) which is in line with previous studies (Wu et al., 2010; Bridwell et al., 2013). Consistent with previous single modality fMRI studies, dynamic EEG-fMRI connectivity shows some connectivity states which re-occur over time. This work provides an important first step in fusing EEG and fMRI using a graph theoretical framework.

In early studies which combine EEG and fMRI, EEG signals are traditionally separated into five frequency bands: delta, theta, alpha, beta, and gamma (Laufs et al., 2003b; Mantini et al., 2007; Keilholz, 2014). Different frequencies have been linked to different functional properties (Buzsaki, 2006). For example, alpha power increases at rest with eyes closed compared to eyes open (Pfurtscheller et al., 1996; de Munck et al., 2007), and increases when a greater number of items are held in working memory (Klimesch et al., 1997, 1999; Jensen et al., 2002). Experimental results have related power increases and synchronization in the gamma frequency band to the performance of perceptual and cognitive operations, including attention (Womelsdorf and Fries, 2007), conscious perception (Melloni et al., 2007), and decision making (Donner et al., 2009). Slower frequencies, such as delta, arise during sleep, and are hypothesized to reflect diminished temporal complexity underlying loss of conscious awareness (Tononi et al., 1994; Tononi and Edelman, 1998; Tononi, 2004, 2008). Low frequency oscillations also appear to correspond to the cognitive events which primarily contribute to evoked potential's (Demiralp et al., 1999).

The majority of previous studies which combine EEG and fMRI data use correlation or general linear modeling (GLM) to link fluctuations between multiple EEG frequency bands and fMRI voxels (Bridwell and Calhoun, 2014). Correlations between EEG power variations of delta, theta, alpha, beta, gamma rhythms and BOLD activity of specific brain regions (ICNs; Laufs et al., 2003b), or BOLD connectivity between brain regions (ICNs) have been estimated (Tagliazucchi et al., 2012). These studies suggest that each functional brain ICN is characterized by a specific electrophysiological signature, and that BOLD fMRI fluctuations have a neurophysiological origin (Mantini et al., 2007).

In this work, we separate the EEG data into five frequency bands as in previous studies. However, in addition to computing the correlations between EEG signals and fMRI BOLD signals, we compute EEG-fMRI multi-modal brain graphs in which EEG nodes provide high temporal resolution information and fMRI nodes provide high spatial resolution. The finding that graph metrics show differences across frequency bands (the main effect of frequency band is significant) is consistent with the hypothesis that different EEG frequencies are associated with different BOLD activities.

Within this study, we characterize different graph properties between eyes open and eyes closed conditions. In static positive connection EEG-fMRI graphs, nodal level graph metrics are higher during the eyes closed condition in three brain components which belong to somatomotor, visual, and auditory areas. In negative connection graphs, a visual component shows different nodal level graph measures (for all three metrics) between eyes conditions. The LFA and VAR of dynamic nodal graph measures of two cognitive control components are higher during eyes closed than eyes open for positive connection networks. In general, these findings are consistent with and add to previous studies demonstrating differences in BOLD amplitudes and functional connectivity across the two conditions (McAvoy et al., 2008; Zou et al., 2009, 2015). Importantly, the present findings provide new insights by incorporating fMRI spatial locations and EEG frequency bands within graph theoretic measures of brain connectivity between eyes closed and eyes open conditions. For example, the differences of graph metrics between eyes conditions are mainly in delta and theta bands for positive connection networks and mainly in alpha and beta bands in negative connection networks.

Multiple recent brain imaging studies suggest that the functional brain connectivity is not stationary but changes over minute-to-minute intervals (Hutchison et al., 2013a; Calhoun et al., 2014). Here we assess dynamic properties (such as LFA and VAR) of the time-varying EEG-fMRI brain graphs and their associated connectivity states. Our results characterize dynamic measures of multi-modal functional brain organization by combining concurrent EEG-fMRI signals, and support the hypothesis that variability of brain connectivity emerges from structured connectivity patterns that emerge and dissolve over time (Allen et al., 2014).

Notably, the findings that alteration of graph measures of specific fMRI nodes across eyes conditions occurs in particular EEG frequency bands provide new electrophysiological signatures of functional brain connectivity examined in fMRI data, and imply that the graph-theory based analysis is powerful to assess the associations between EEG and fMRI. However, a few potential methodological limitations need to be discussed. Graph metrics may depend in part on the methods used to identify nodes. Thus, it is worth considering the difference between ICA-based and anatomically-based approaches. Within fMRI, brain graph nodes are often formed using predefined anatomical templates such as automated anatomical labeling (AAL; Tzourio-Mazoyer et al., 2002; Liu et al., 2008; Lynall et al., 2010), randomly generated templates (Hagmann et al., 2008; Fornito et al., 2010), and voxel-based divisions (Eguíluz et al., 2005; Buckner et al., 2009; Yu et al., 2011c, 2013). Different approaches may significantly modulate the quantitative measures of graph metrics of brain connectivity (Fornito et al., 2013; de Reus and van den Heuvel, 2013). Also, prior work has shown a detriment to network estimation when using atlas-based regions of interest (ROIs) as graph nodes (Smith et al., 2011; Craddock et al., 2012; Shirer et al., 2012). Moreover, the ROIs provide an imperfect segregation of the functional boundaries of the human brain. However, ICA, which is adopted in this study, provides a data-driven approach to identify spatial brain components as functionally homogeneous nodes (Yu et al., 2011a,b; Calhoun and Adali, 2012; Calhoun et al., 2012). We choose a relatively high model order (i.e., 100 ICs) ICA, because previous studies have demonstrated that such models yield refined components which correspond to known anatomical and functional segmentations (Kiviniemi et al., 2009; Abou-Elseoud et al., 2010). Importantly, previous work has shown that fMRI graph measures are relatively insensitive to high model orders (Yu et al., 2011b, 2015). However, a limitation of this study is related to the differences in node number and edge weight from different modalities. Positive correlations within EEG signals are much higher than within fMRI signals and between EEG-fMRI signals (see Figure 2B). Also, the Pearson correlation may contain some redundant information, though the ICA performed in data processing, which fits all the interacting network information in a single model with multiple components, may control some of them.

It's important to note that graph measures were computed based on the formula defined for a single-modal (classical) graph. The graph metrics may be interpreted in the same way as traditional graphs. But it is unclear how global level graph measures within a multi-modal graph would be affected by the distribution of edges and nodes from different modalities. This limitation is shared by each of the two conditions examined within the present study (eyes open and eyes closed). Thus, the observation of graph metric differences here motivates further studies which extend the single-modal (classical) graph formula for EEG-fMRI (Zhang et al., 2008). In addition, future work is needed to develop criteria for determining the number of nodes and edges in the context of a multi-modal brain graph. Also, future work may build EEG and fMRI graphs separately and evaluate the correlation between graph metrics between the two, or develop new methods for defining brain regions (graph nodes) with both EEG and fMRI information available as previous studies which estimated brain graph using multi-modality imaging data(He et al., 2008; Hermundstad et al., 2013; Liang et al., 2013; van den Heuvel and Sporns, 2013a; Tewarie et al., 2014a,b).

## Conclusions

We believe that this work provides an important beginning step in characterizing EEG-fMRI associations within a graph theoretical framework. Both static and dynamic EEG-fMRI graphs are built in five EEG frequency bands on concurrently collected EEG-fMRI data while individuals rested with eyes open and eyes closed. Differences in global and nodal level static graph metrics including connectivity strength, local efficiency, and global efficiency, are revealed among frequency bands and between eyes conditions. Dynamic properties of the graph metrics also show differences between eyes conditions. These findings incorporate spatial location (provided by fMRI) information and frequency (delta, theta, alpha, beta, and gamma bands provided by EEG) information in identifying graph properties that differ between brain states (i.e., eyes open vs. eyes closed) by linking electro-hemodynamic responses. This paper proposes a novel approach for assessing associations among concurrent EEG and fMRI measures which couples electoral and hemodynamic BOLD signals in the brain at a network level.

## Author contributions

QY designed the study; analyzed and interpreted the data; drafted and revised the manuscript; gave final approval. LW designed the study; collected, analyzed, and interpreted the data; revised the manuscript and gave final approval. DB analyzed and interpreted the data; revised the manuscript and gave final approval. EE analyzed and interpreted the data; revised the manuscript and gave final approval. YD analyzed and interpreted the data; revised the manuscript and gave final approval. HH analyzed and interpreted the data; revised the manuscript and gave final approval. JC collected, analyzed, and interpreted the data; revised the manuscript. PL analyzed and interpreted the data; revised the manuscript and gave final approval. JS collected, analyzed, and interpreted the data; revised the manuscript. GP designed the study; interpreted the data; revised the manuscript and gave final approval. VC designed the study; interpreted the data; revised the manuscript and gave final approval.

### Conflict of interest statement

The authors declare that the research was conducted in the absence of any commercial or financial relationships that could be construed as a potential conflict of interest.
