# Supplementary material for: Building an EEG-fMRI Multi-Modal Brain Graph: A Concurrent EEG-fMRI Study
Source: Front Hum Neurosci. 2016 Sep 28;10:476. doi: 10.3389/fnhum.2016.00476 (PMC5039193; doi:10.3389/fnhum.2016.00476)
Supplement: Supplementary file 1 [file Table1.DOCX]

**Table S1.** Number of connectivity states detected in each subject in all five frequency bands during eyes open and eyes closed for negative networks.

| **Subject ID** | **delta** | | **theta** | | **alpha** | | **beta** | | **gamma** | |
| --- | --- | --- | --- | --- | --- | --- | --- | --- | --- | --- |
|  | **eyes open** | **eyes close** | **eyes open** | **eyes close** | **eyes open** | **eyes close** | **eyes open** | **eyes close** | **eyes open** | **eyes close** |
| **S1** | 3 | 6 | 5 | 4 | 4 | 4 | 4 | 3 | 4 | 5 |
| **S2** | 4 | 4 | 4 | 3 | 4 | 2 | 4 | 3 | 4 | 3 |
| **S3** | 2 | 4 | 4 | 4 | 4 | 5 | 4 | 5 | 3 | 4 |
| **S4** | 6 | 3 | 5 | 2 | 3 | 3 | 5 | 3 | 3 | 3 |
| **S5** | 4 | 3 | 6 | 3 | 4 | 4 | 4 | 4 | 3 | 4 |
| **S6** | 4 | 4 | 4 | 4 | 3 | 3 | 3 | 4 | 4 | 4 |
| **S7** | 6 | 3 | 2 | 3 | 4 | 4 | 4 | 3 | 5 | 5 |
| **S8** | 3 | 3 | 4 | 3 | 6 | 6 | 4 | 4 | 5 | 4 |
| **S9** | 4 | 4 | 4 | 4 | 4 | 5 | 4 | 3 | 4 | 4 |
| **S10** | 4 | 3 | 4 | 3 | 4 | 3 | 4 | 3 | 4 | 3 |
| **S11** | 5 | 2 | 5 | 4 | 3 | 3 | 5 | 3 | 3 | 4 |
| **S12** | 4 | 4 | 3 | 4 | 4 | 4 | 4 | 4 | 5 | 5 |
| **S13** | 5 | 4 | 3 | 4 | 4 | 3 | 5 | 3 | 4 | 5 |
| **S14** | 3 | 2 | 6 | 2 | 3 | 4 | 4 | 3 | 5 | 4 |
| **S15** | 4 | 4 | 4 | 5 | 4 | 4 | 3 | 4 | 5 | 4 |
| **S16** | 4 | 5 | 4 | 3 | 4 | 4 | 3 | 5 | 3 | 3 |
| **S17** | 5 | 4 | 4 | 4 | 4 | 4 | 6 | 4 | 4 | 5 |
| **S18** | 3 | 4 | 3 | 3 | 3 | 4 | 3 | 4 | 3 | 4 |
| **S19** | 4 | 4 | 3 | 3 | 2 | 6 | 4 | 3 | 3 | 4 |
| **S20** | 3 | 3 | 4 | 4 | 3 | 5 | 3 | 4 | 5 | 4 |
| **S21** | 3 | 3 | 3 | 4 | 4 | 4 | 4 | 4 | 4 | 3 |
| **S22** | 4 | 5 | 3 | 3 | 5 | 4 | 4 | 3 | 5 | 3 |
| **S23** | 4 | 3 | 3 | 4 | 3 | 3 | 3 | 5 | 3 | 5 |
| **S24** | 4 | 5 | 6 | 2 | 3 | 3 | 3 | 4 | 4 | 4 |
| **S25** | 4 | 4 | 3 | 5 | 3 | 4 | 4 | 5 | 6 | 4 |
| **total** | 99 | 93 | 99 | 87 | 92 | 98 | 98 | 93 | 101 | 100 |

**Table S2.** Number of connectivity states detected in each subject in all five frequency bands during eyes open and eyes closed for positive networks.

| **Subject ID** | **delta** | | **theta** | | **alpha** | | **beta** | | **gamma** | |
| --- | --- | --- | --- | --- | --- | --- | --- | --- | --- | --- |
|  | **eyes open** | **eyes close** | **eyes open** | **eyes close** | **eyes open** | **eyes close** | **eyes open** | **eyes close** | **eyes open** | **eyes close** |
| **S1** | 5 | 4 | 3 | 3 | 4 | 3 | 3 | 6 | 4 | 3 |
| **S2** | 4 | 3 | 4 | 4 | 4 | 4 | 4 | 5 | 3 | 3 |
| **S3** | 3 | 4 | 4 | 3 | 3 | 3 | 3 | 4 | 4 | 4 |
| **S4** | 4 | 3 | 5 | 3 | 4 | 3 | 3 | 3 | 3 | 4 |
| **S5** | 4 | 2 | 3 | 4 | 2 | 4 | 3 | 3 | 3 | 4 |
| **S6** | 4 | 3 | 4 | 5 | 4 | 4 | 4 | 3 | 6 | 2 |
| **S7** | 3 | 3 | 2 | 4 | 3 | 2 | 3 | 3 | 5 | 3 |
| **S8** | 4 | 4 | 3 | 3 | 3 | 4 | 5 | 4 | 4 | 2 |
| **S9** | 4 | 4 | 3 | 3 | 4 | 3 | 2 | 4 | 3 | 3 |
| **S10** | 2 | 4 | 3 | 3 | 3 | 2 | 3 | 2 | 4 | 3 |
| **S11** | 3 | 3 | 3 | 3 | 4 | 2 | 3 | 2 | 3 | 3 |
| **S12** | 4 | 3 | 3 | 5 | 4 | 4 | 5 | 6 | 4 | 6 |
| **S13** | 4 | 2 | 3 | 4 | 4 | 5 | 3 | 4 | 4 | 3 |
| **S14** | 3 | 3 | 4 | 3 | 4 | 3 | 2 | 5 | 4 | 3 |
| **S15** | 4 | 6 | 2 | 4 | 4 | 3 | 3 | 4 | 5 | 2 |
| **S16** | 4 | 3 | 3 | 4 | 3 | 4 | 4 | 3 | 3 | 2 |
| **S17** | 4 | 4 | 3 | 4 | 3 | 4 | 5 | 3 | 5 | 2 |
| **S18** | 6 | 3 | 3 | 3 | 4 | 2 | 3 | 2 | 3 | 2 |
| **S19** | 4 | 3 | 2 | 3 | 3 | 3 | 4 | 4 | 5 | 3 |
| **S20** | 4 | 3 | 5 | 4 | 4 | 3 | 3 | 3 | 3 | 2 |
| **S21** | 2 | 4 | 2 | 3 | 4 | 3 | 3 | 2 | 4 | 5 |
| **S22** | 3 | 3 | 4 | 2 | 2 | 3 | 4 | 3 | 3 | 4 |
| **S23** | 3 | 4 | 4 | 3 | 4 | 3 | 4 | 4 | 3 | 3 |
| **S24** | 2 | 5 | 6 | 2 | 2 | 4 | 3 | 3 | 5 | 4 |
| **S25** | 4 | 3 | 3 | 2 | 3 | 3 | 3 | 3 | 5 | 2 |
| **total** | 91 | 86 | 84 | 84 | 86 | 81 | 85 | 88 | 98 | 77 |
