# Supplementary material for: Building an EEG-fMRI Multi-Modal Brain Graph: A Concurrent EEG-fMRI Study
Source: Front Hum Neurosci. 2016 Sep 28;10:476. doi: 10.3389/fnhum.2016.00476 (PMC5039193; doi:10.3389/fnhum.2016.00476)
Supplement: Supplementary file 2 [file Image1.PDF]

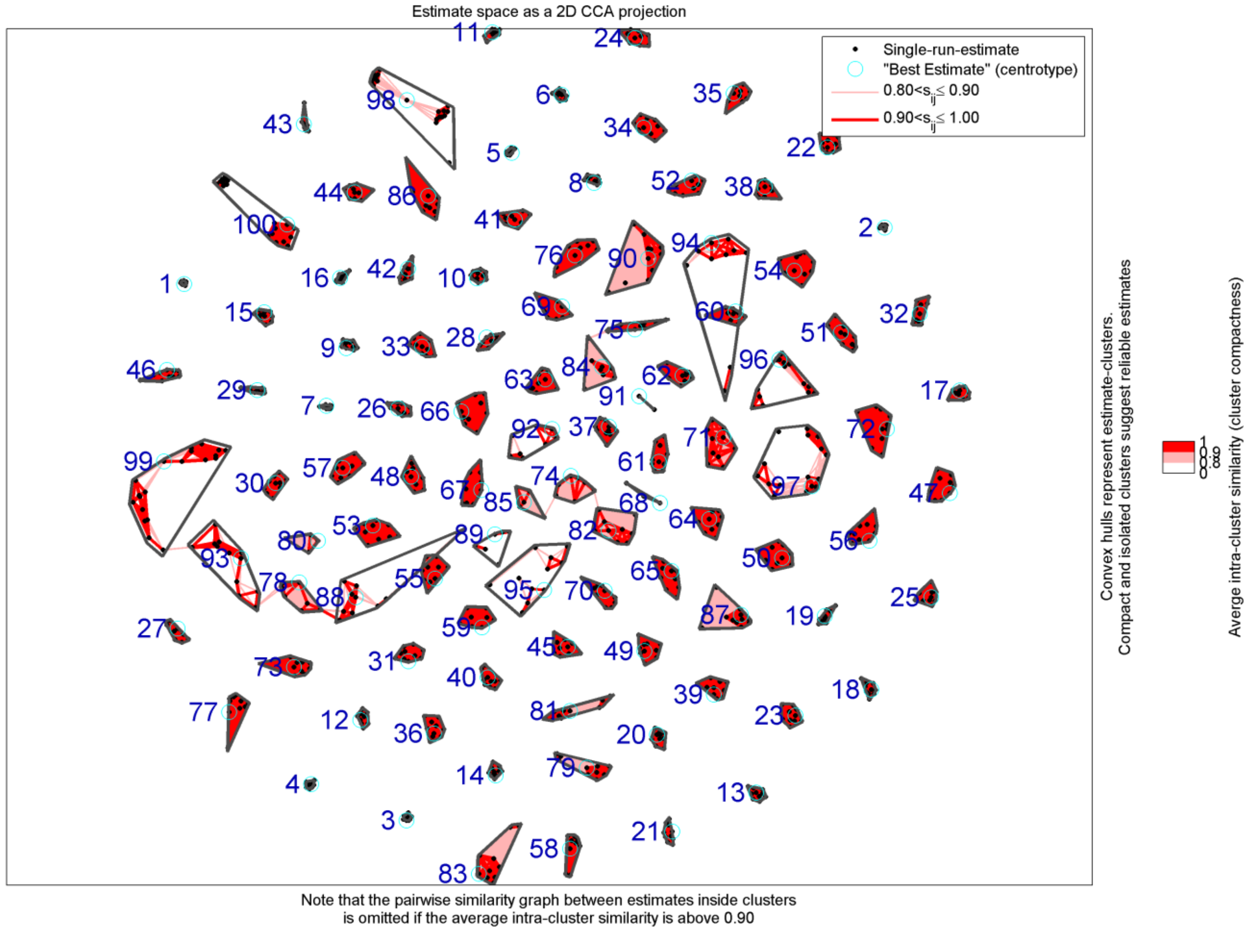

Figure S1. Similarity graph of the fMRI brain components output from ICASSO.

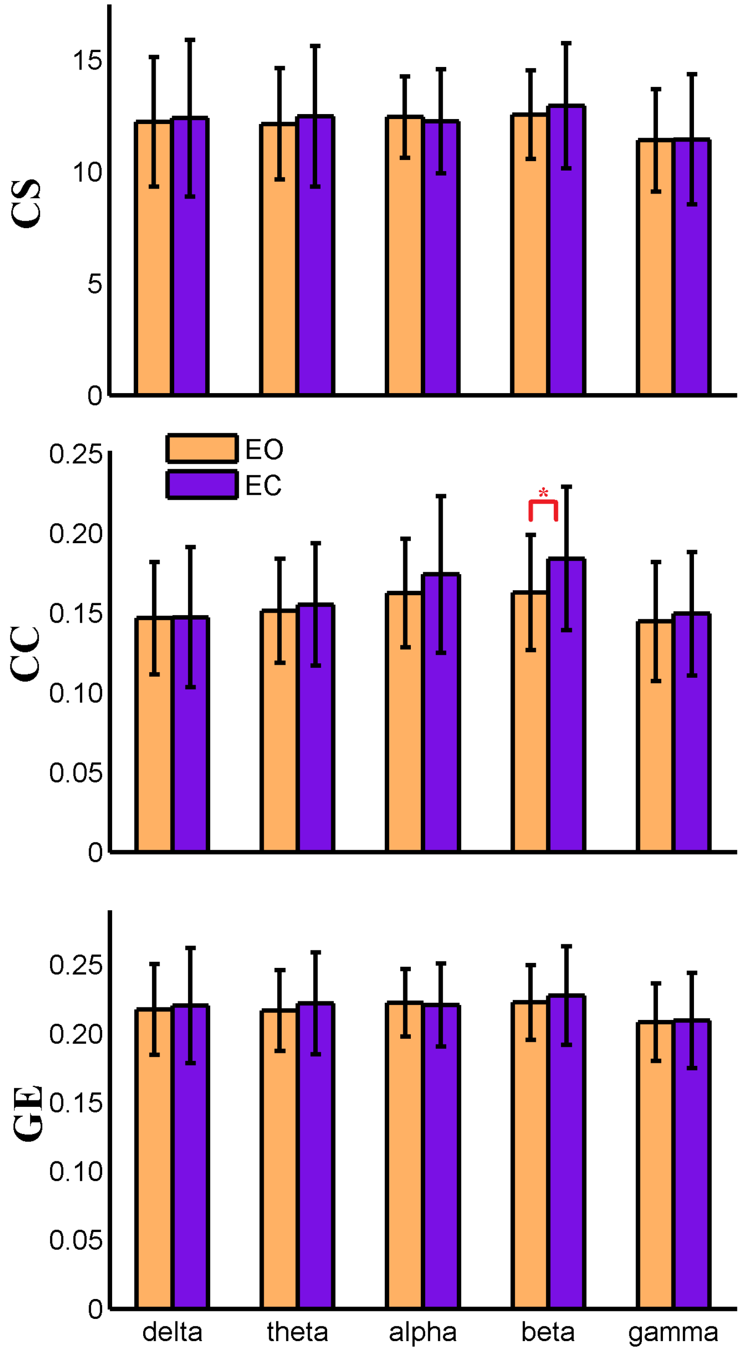

Figure S2. Mean value of global level graph metrics (CS: connectivity strength; CC: clustering coefficient; GE: global efficiency) for static positive connection graphs. The color of the bar indicates the eyes condition and the height of the bar indicates the mean value of the measurement for the 25 subjects. Error bars correspond to standard deviation. Statistical analysis shows that the main effects of frequency band is significant for all three metrics ($P < 0.001$), and the main effect of eyes condition is significant ($P < 0.05$) for CC. ('*' indicates $P < 0.01$ for posthoc paired t-test).

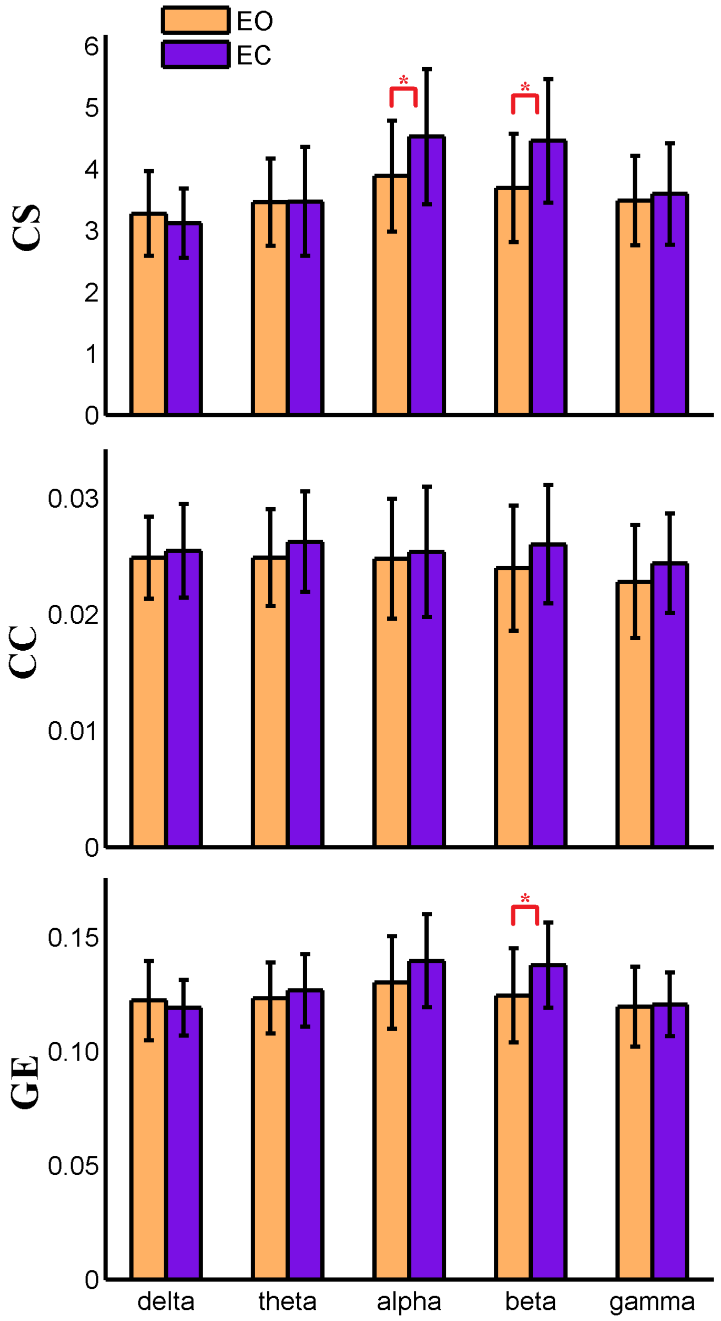

Figure S3. Mean value of global level graph metrics (CS: connectivity strength; CC: clustering coefficient; GE: global efficiency) for static negative connection graphs. The color of the bar indicates the eyes condition and the height of the bar indicates the mean value of the measurement for the 25 subjects. Error bars correspond to standard deviation. Statistical analysis shows that the main effect of frequency band is significant for CS and CC ($P < 0.05$), and the main effect of eyes condition is significant ($P < 0.01$) for all of the three metrics.

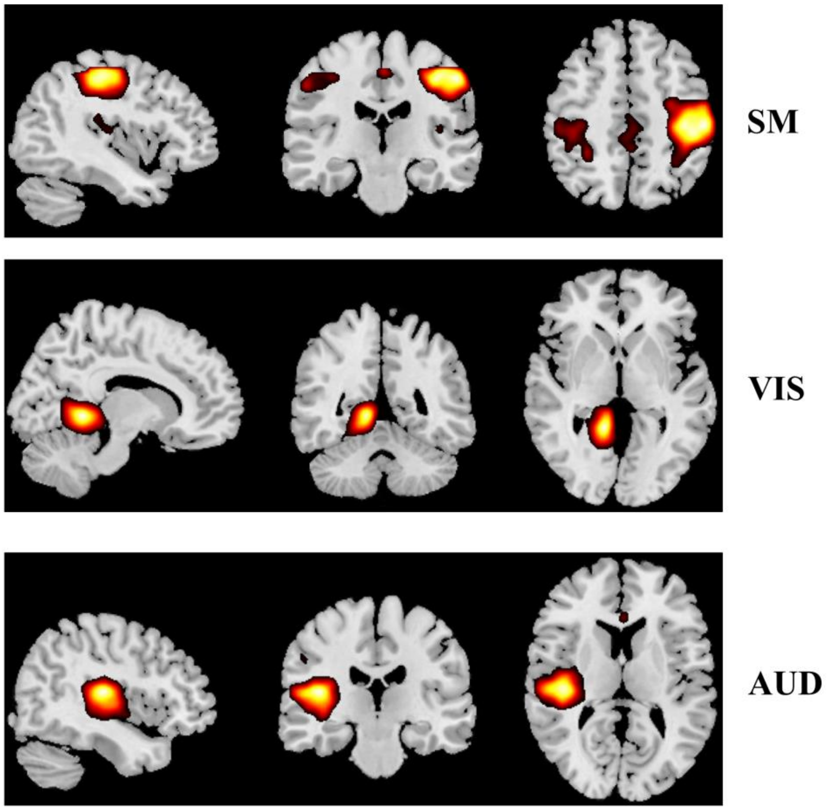

Figure S4. Three fMRI brain components which show significant higher ($P < 0.001$, FDR correction) values of all three graph metrics in the static positive connection graphs in eyes closed than in eyes open. They belongs to auditory, somatomotor, and visual components respectively,

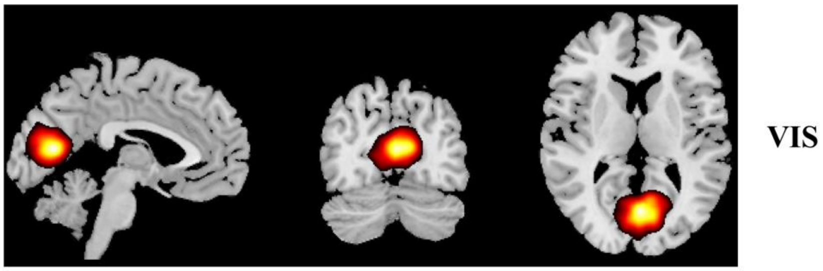

Figure S5. One brain component which shows significant ($P < 0.001$, FDR correction) higher values of graph metrics in the static graph in eyes closed than in eyes open for the negative connection graph. It belongs to visual components,

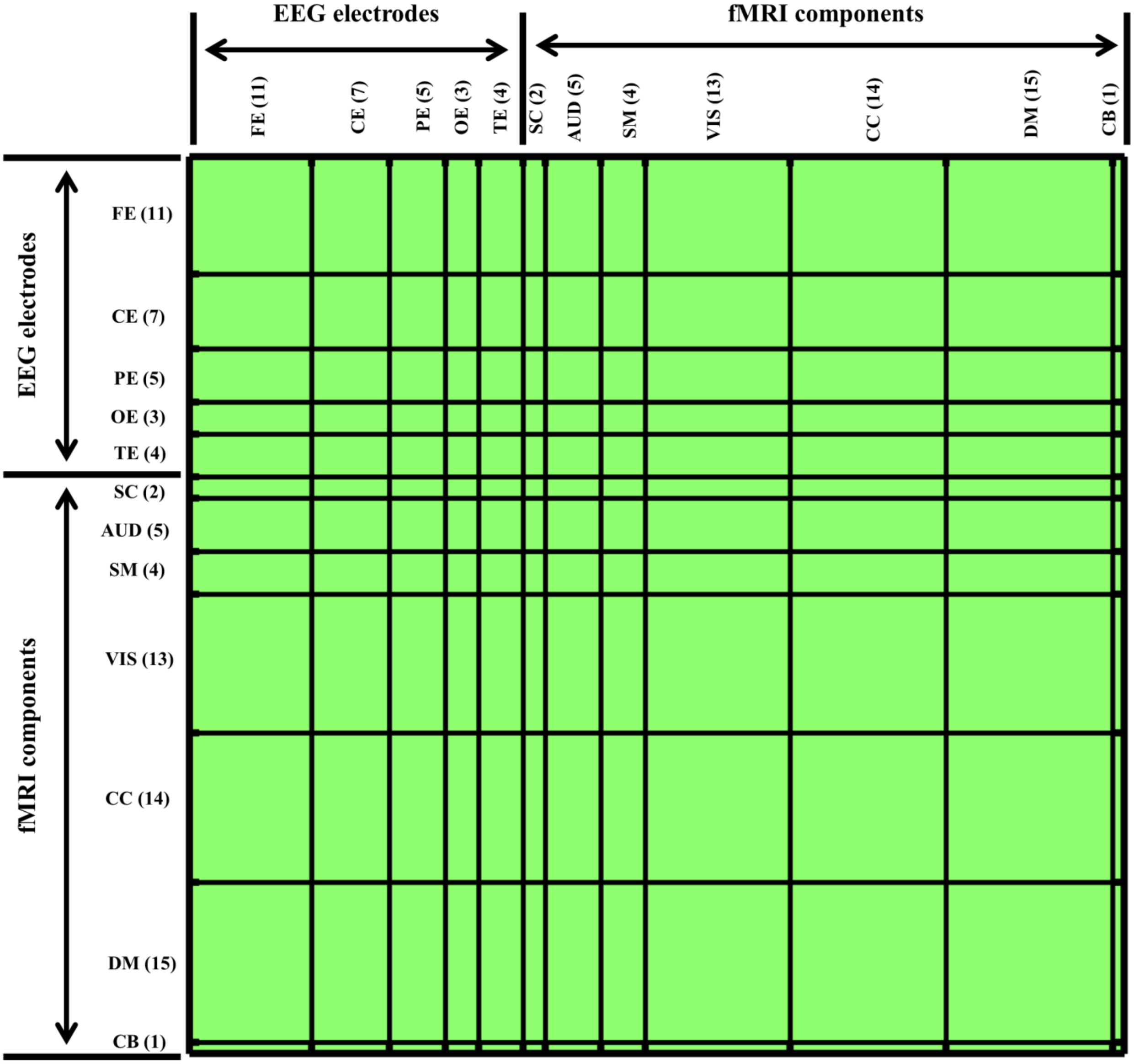

Figure S6. Nodes organization for the EEG-fMRI graph figures in this paper.

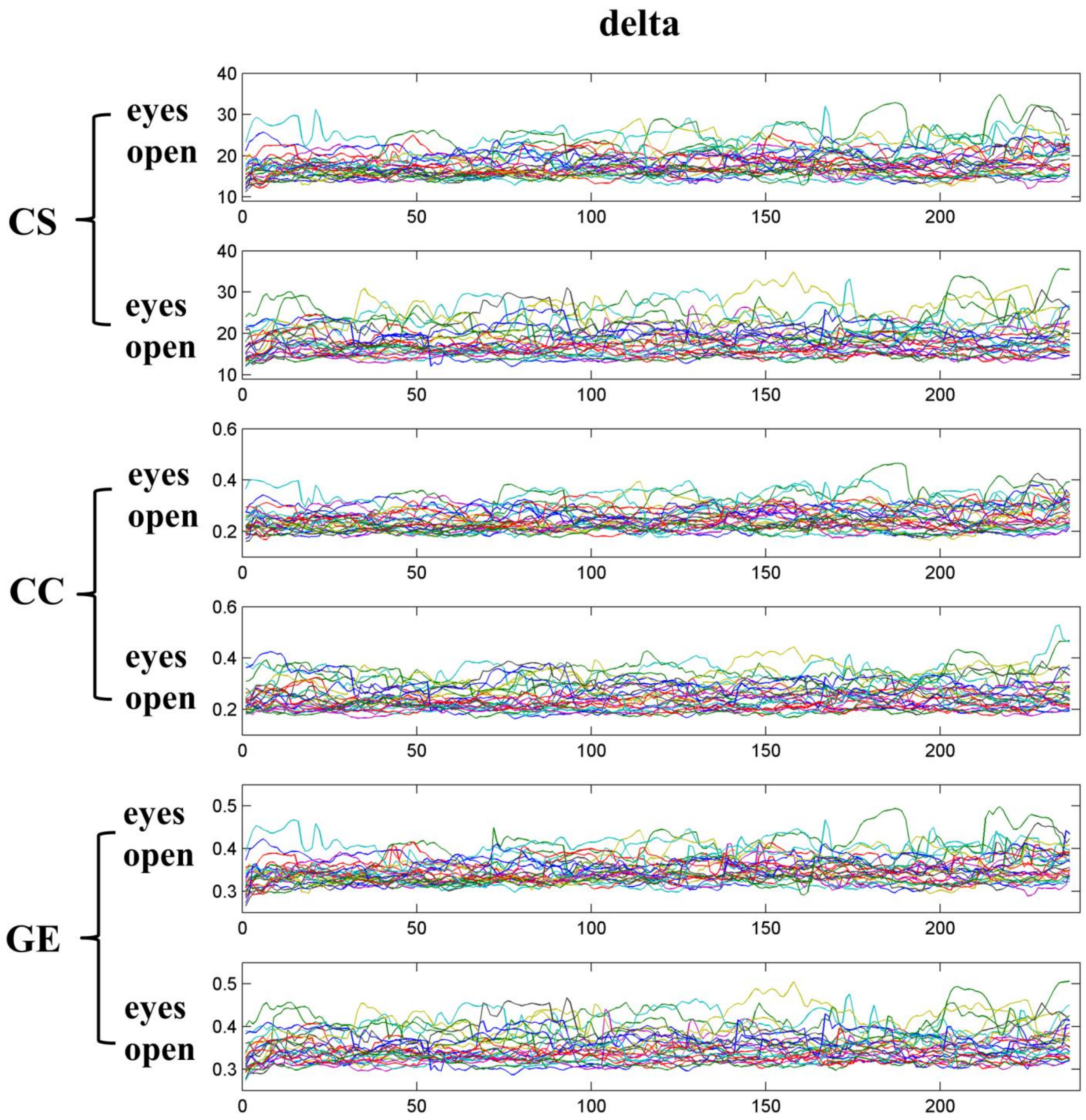

Figure S7. Global level graph metrics (CS: connectivity strength; CC: clustering coefficient; GE: global efficiency) of the time varying positive connection EEG-fMRI graphs for delta frequency band (over 237 time windows; x is the index of time windows). Graph measures appear to dynamically change over time.

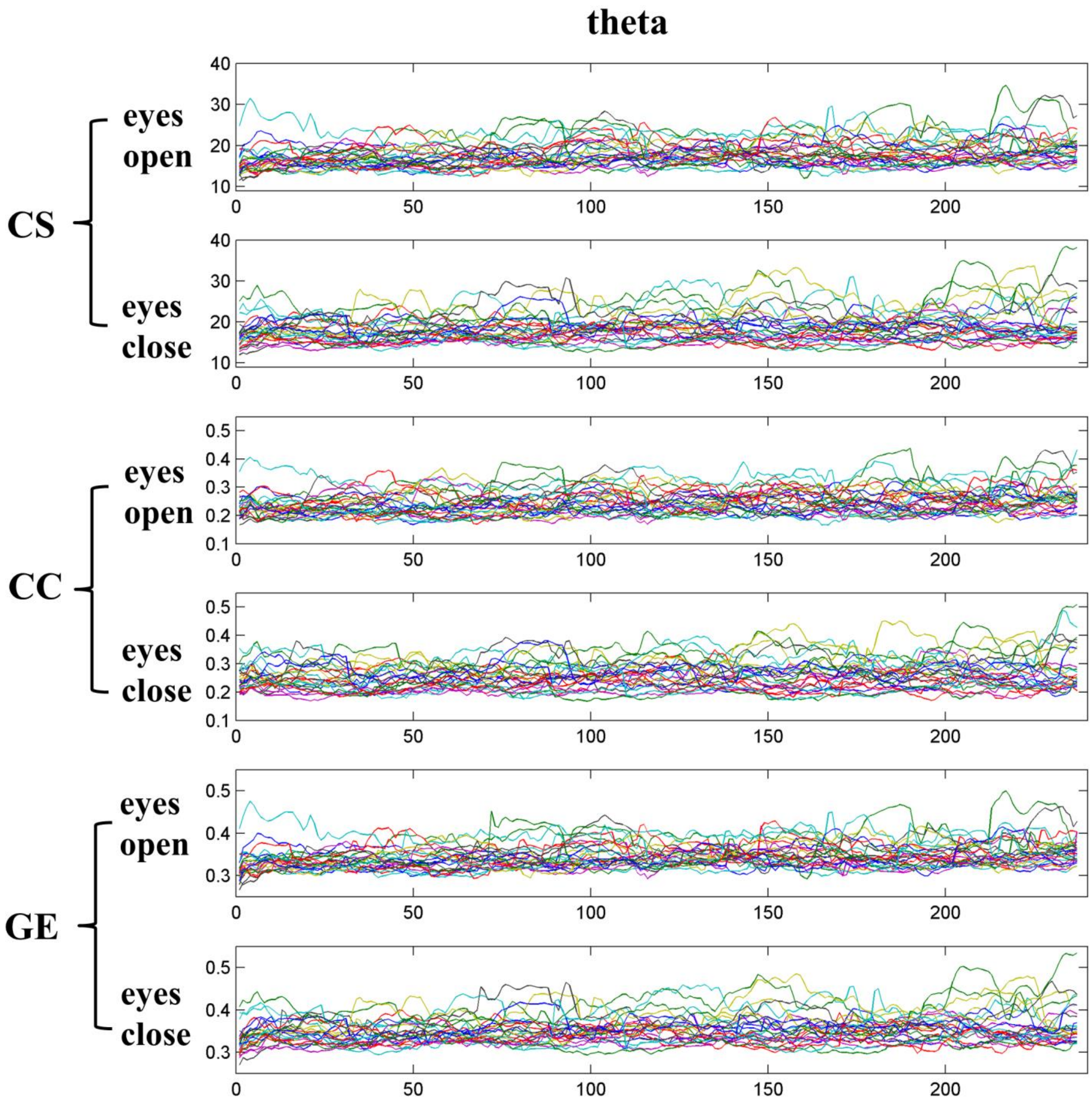

Figure S8. Global level graph metrics (CS: connectivity strength; CC: clustering coefficient; GE: global efficiency) of the time varying positive connection EEG-fMRI graphs for theta frequency band (over 237 time windows; x is the index of time windows). Graph measures appear to dynamically change over time.

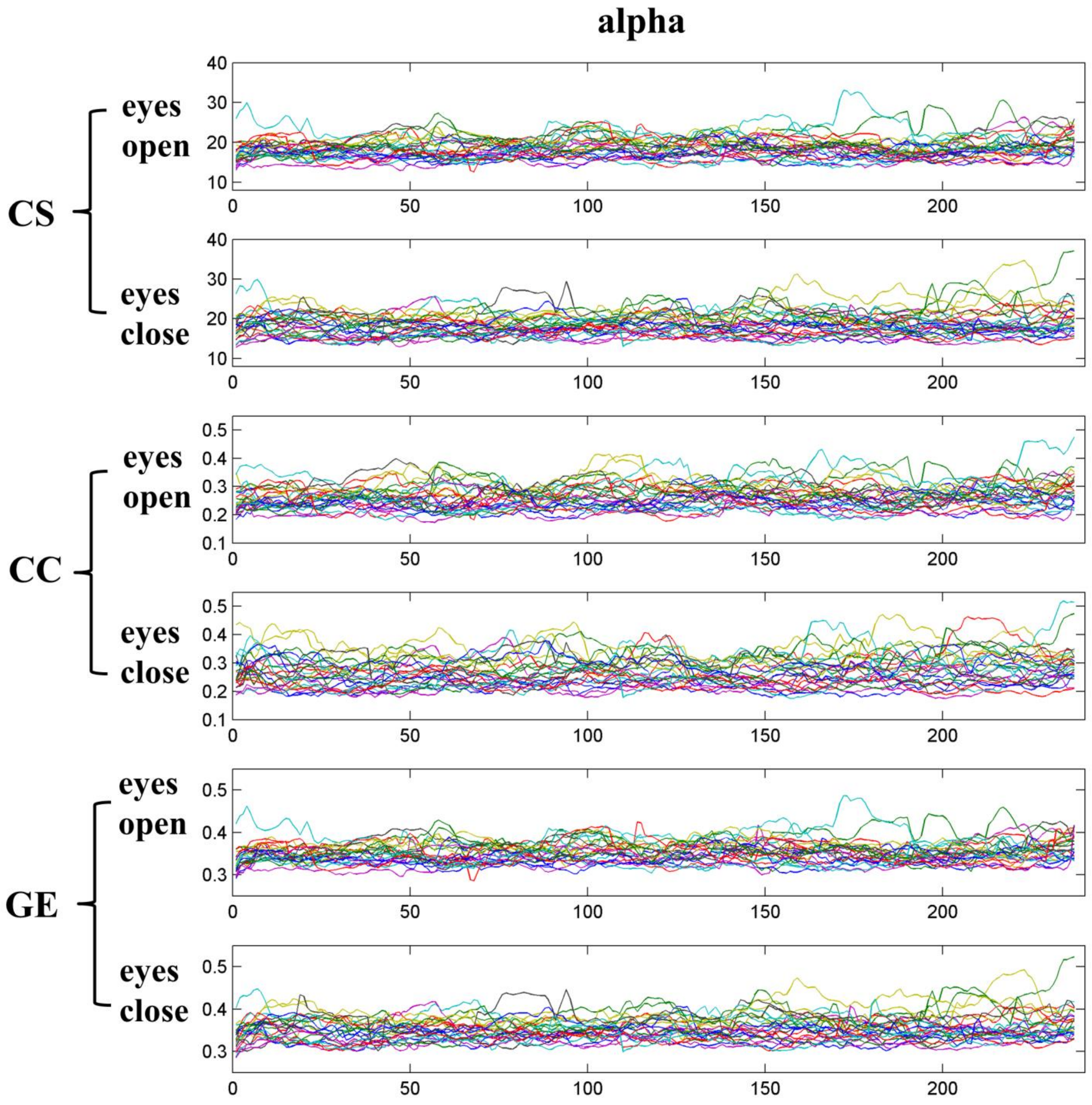

Figure S9. Global level graph metrics (CS: connectivity strength; CC: clustering coefficient; GE: global efficiency) of the time varying positive connection EEG-fMRI graphs for alpha frequency band (over 237 time windows; x is the index of time windows). Graph measures appear to dynamically change over time.

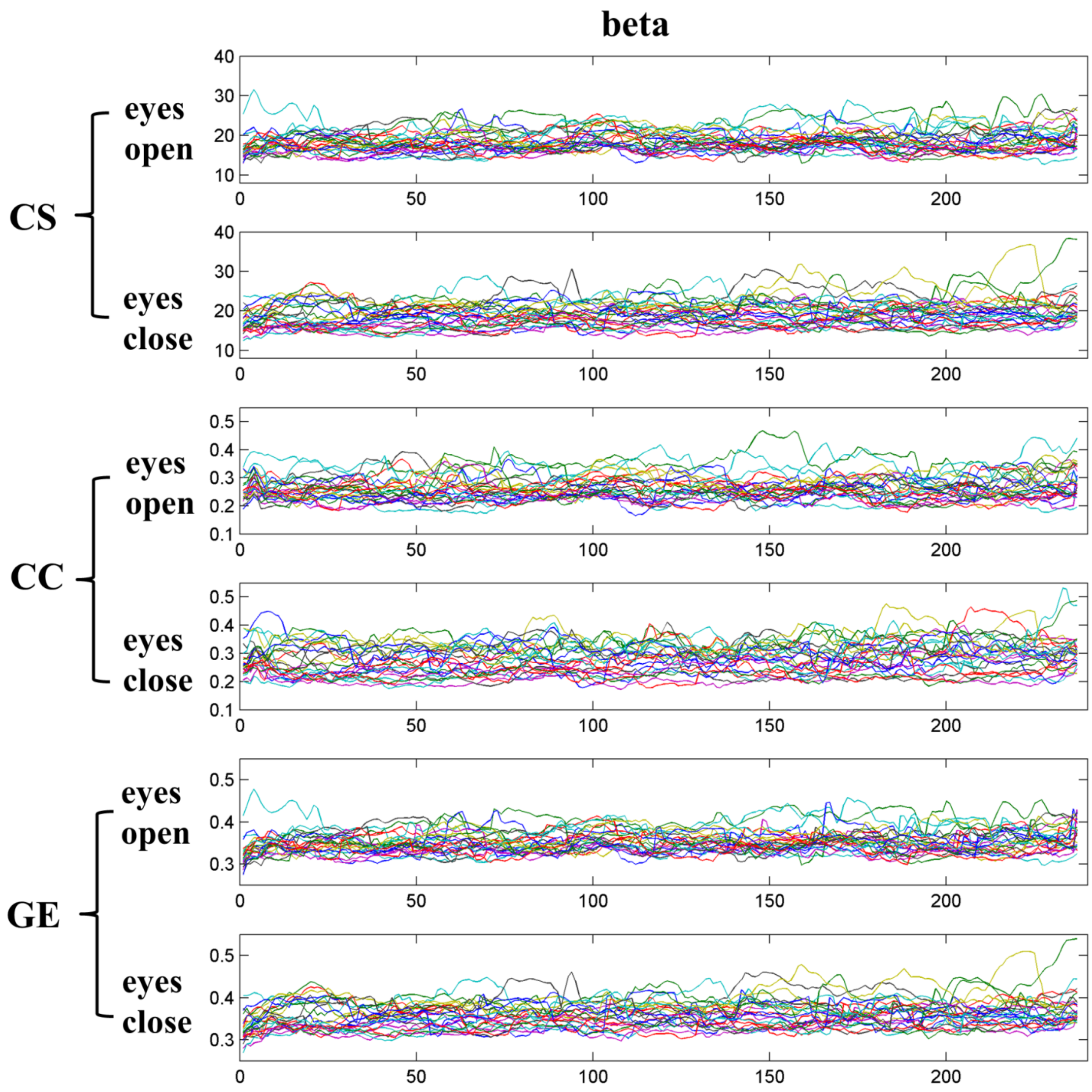

Figure S10. Global level graph metrics (CS: connectivity strength; CC: clustering coefficient; GE: global efficiency) of the time varying positive connection EEG-fMRI graphs for beta frequency band (over 237 time windows; x is the index of time windows). Graph measures appear to dynamically change over time.

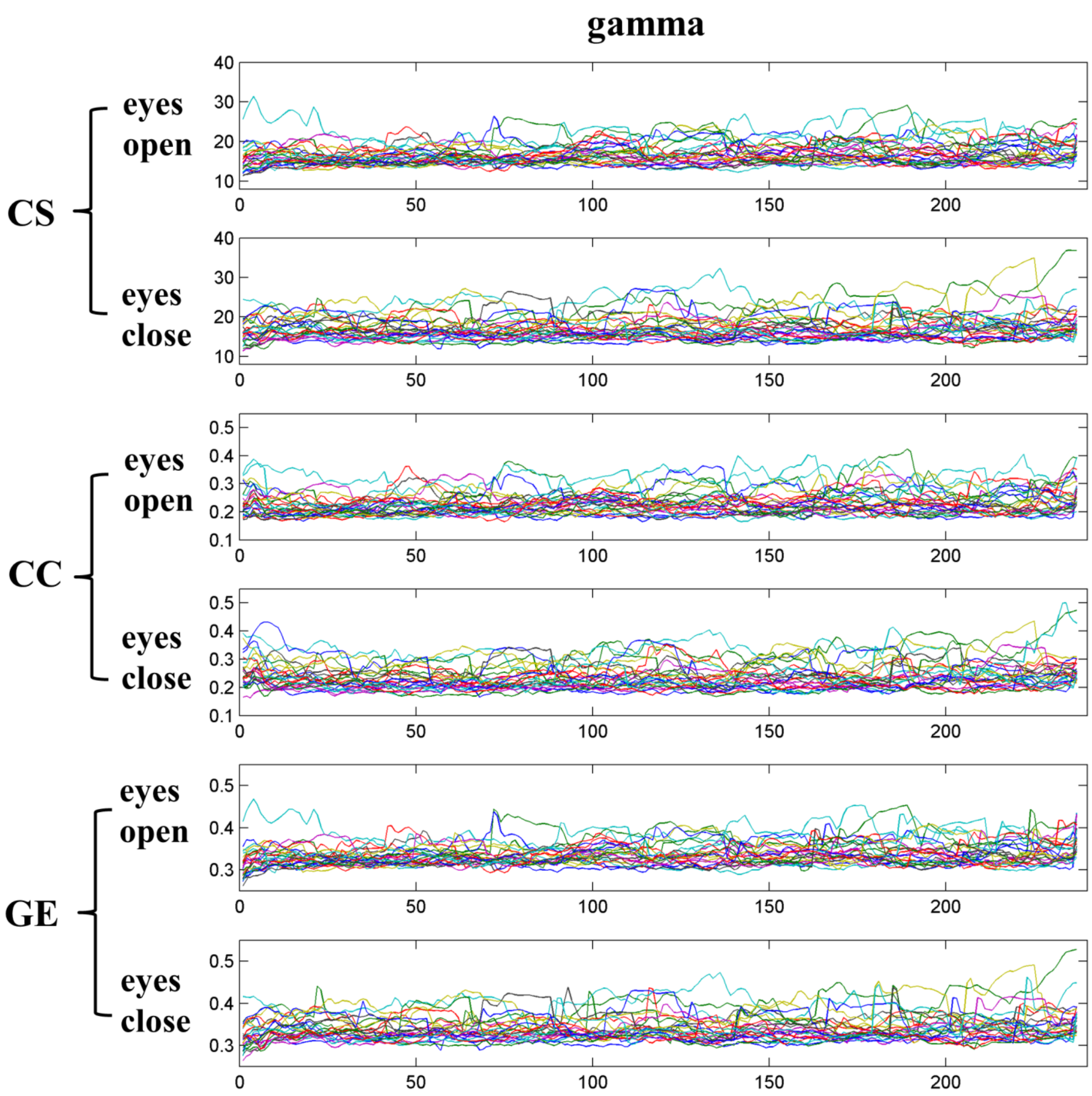

Figure S11. Global level graph metrics (CS: connectivity strength; CC: clustering coefficient; GE: global efficiency) of the time varying positive connection EEG-fMRI graphs for low gamma frequency band (over 237 time windows; x is the index of time windows). Graph measures appear to dynamically change over time.

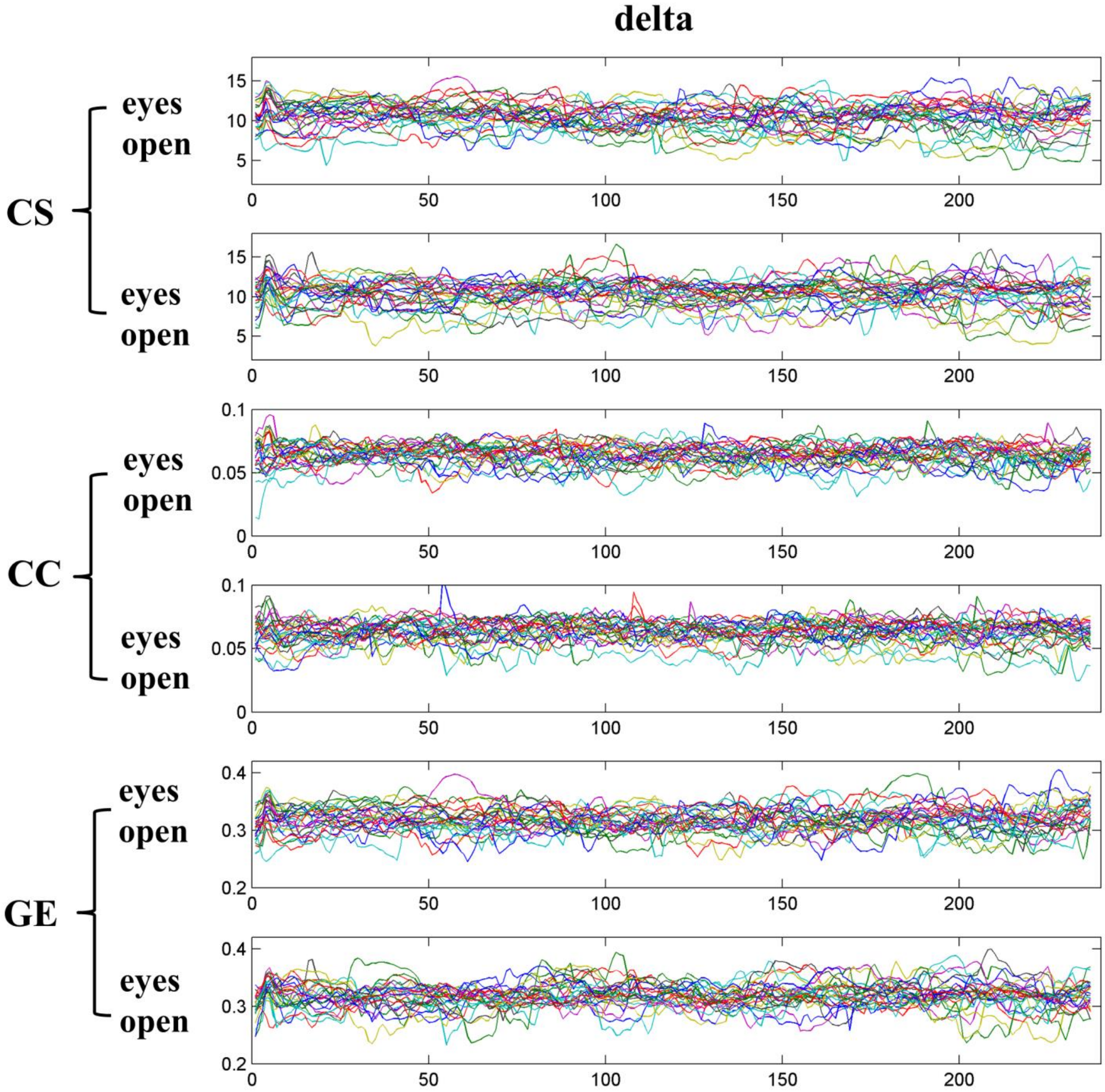

Figure S12. Global level graph metrics (CS: connectivity strength; CC: clustering coefficient; GE: global efficiency) of the time varying negative connection EEG-fMRI graphs for delta frequency band (over 237 time windows; x is the index of time windows). Graph measures appear to dynamically change over time.

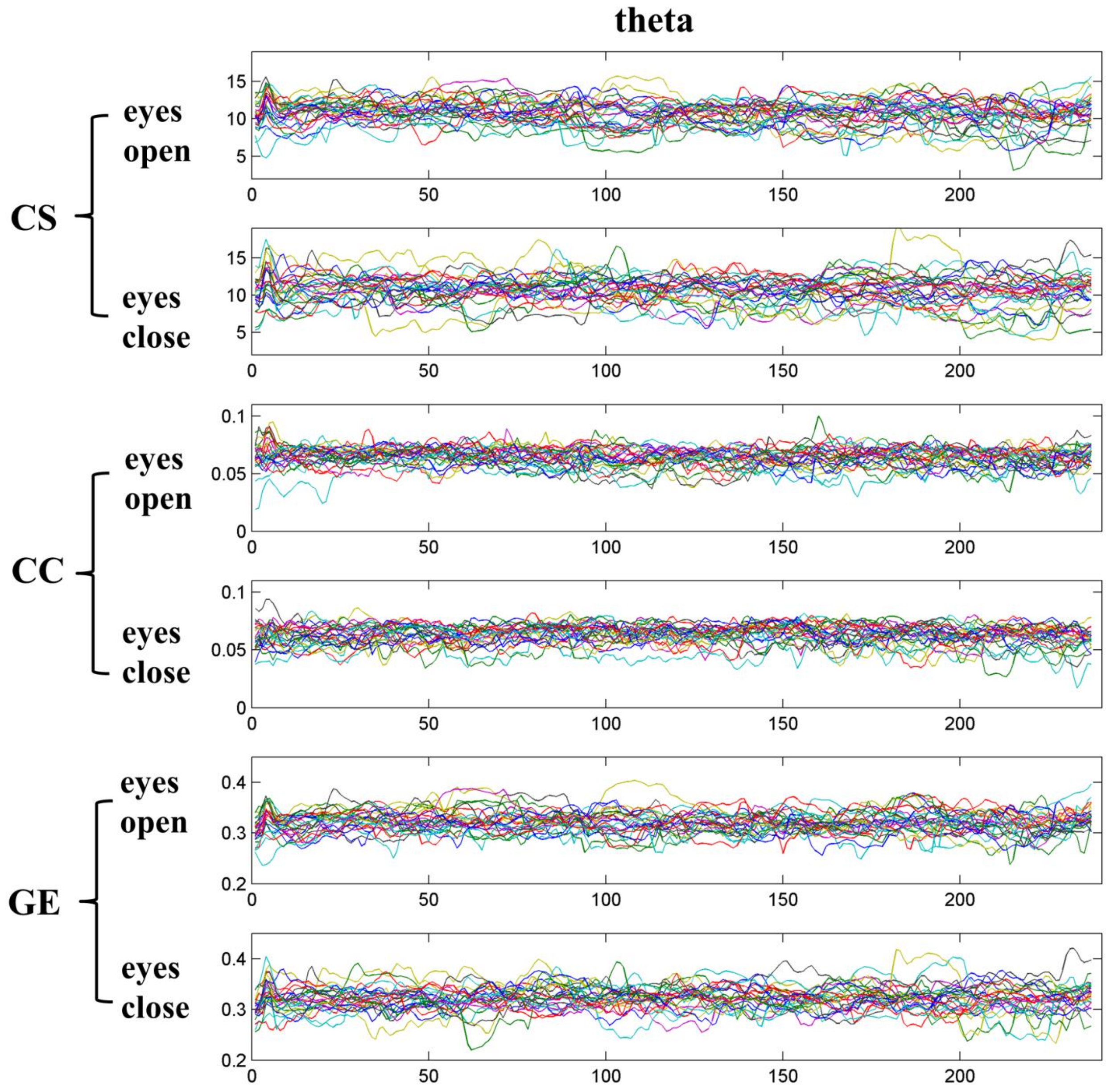

Figure S13. Global level graph metrics (CS: connectivity strength; CC: clustering coefficient; GE: global efficiency) of the time varying negative connection EEG-fMRI graphs for theta frequency band (over 237 time windows; x is the index of time windows). Graph measures appear to dynamically change over time.

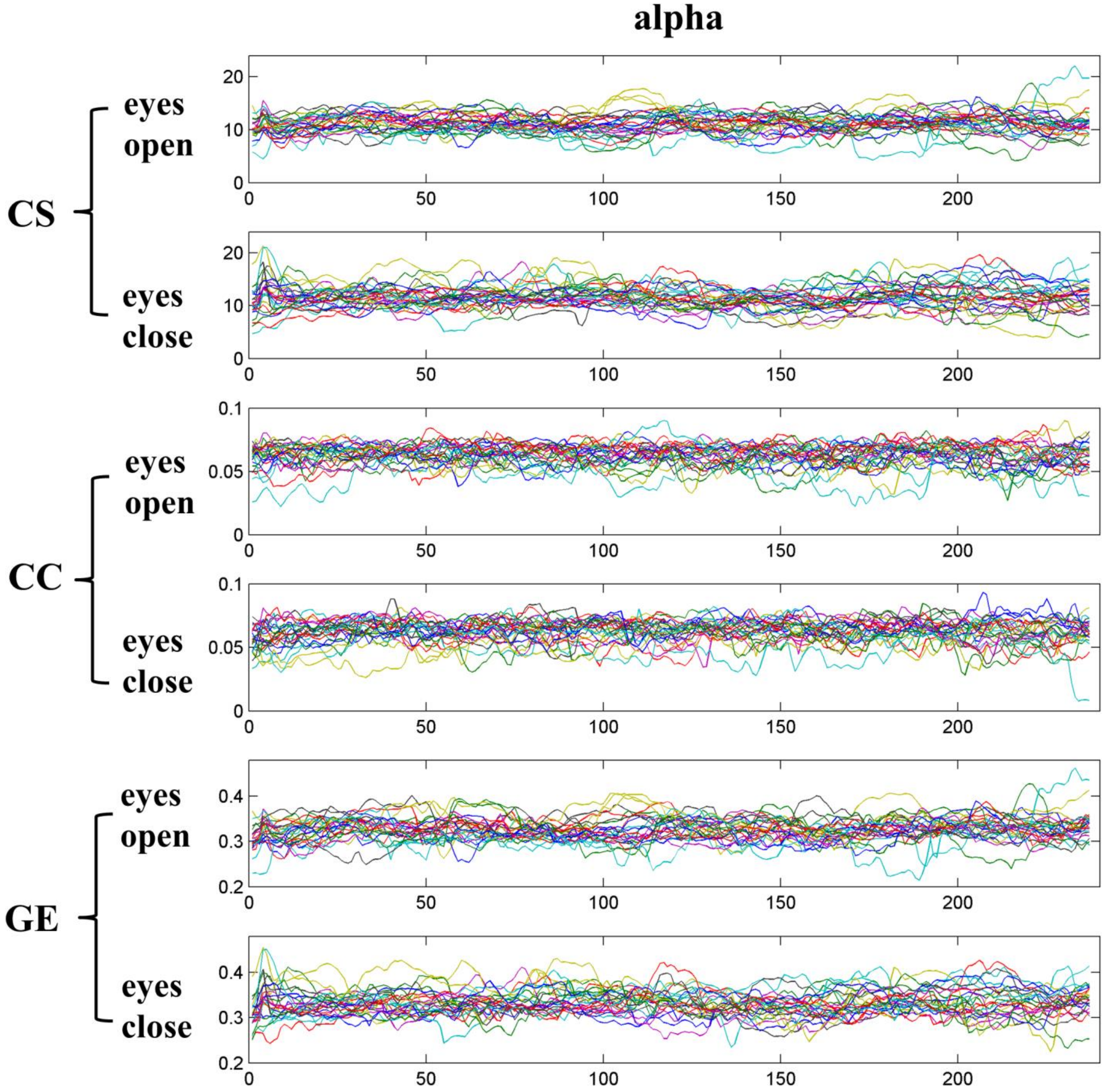

Figure S14. Global level graph metrics (CS: connectivity strength; CC: clustering coefficient; GE: global efficiency) of the time varying negative connection EEG-fMRI graphs for alpha frequency band (over 237 time windows; x is the index of time windows). Graph measures appear to dynamically change over time.

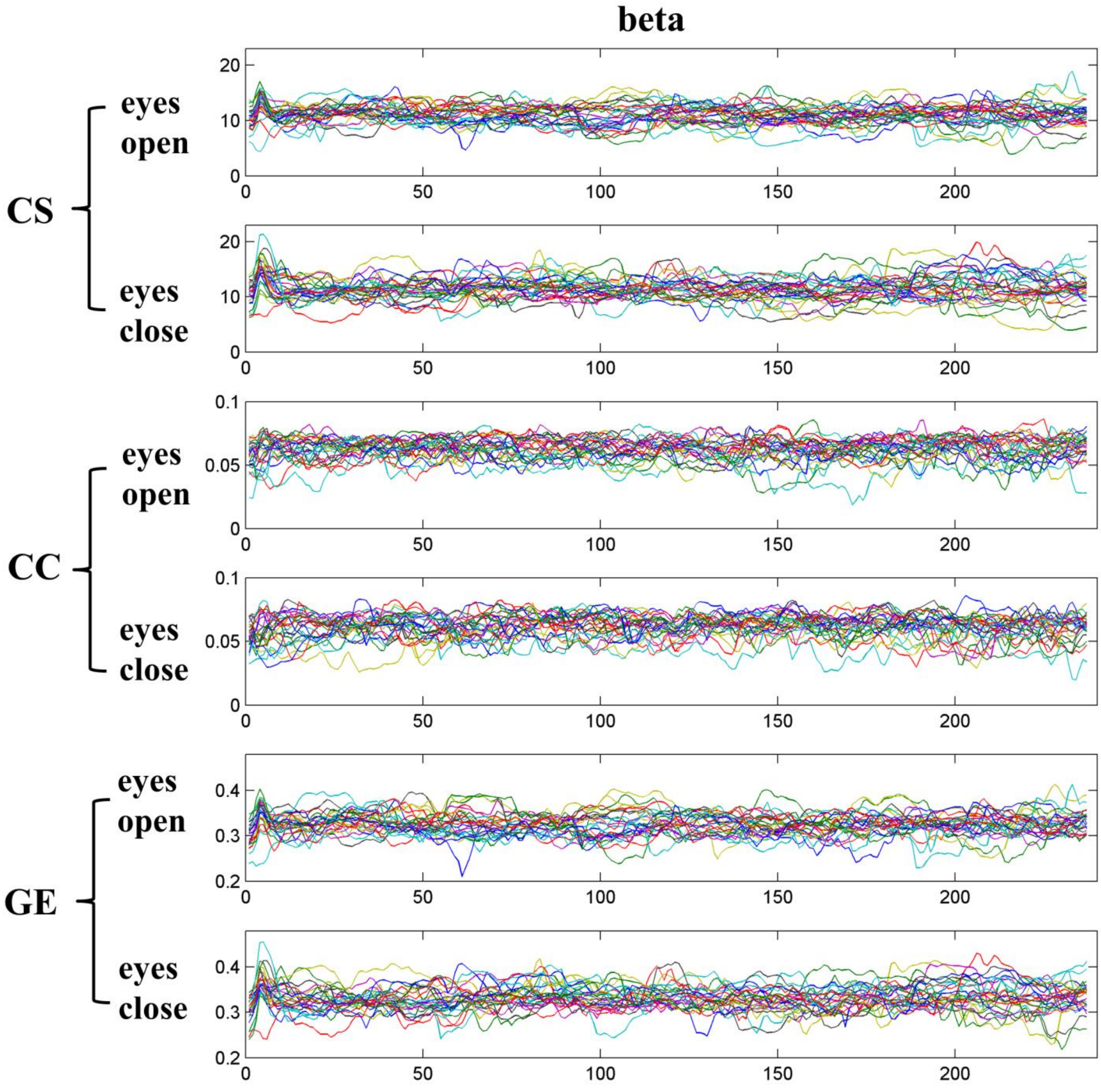

Figure S15. Global level graph metrics (CS: connectivity strength; CC: clustering coefficient; GE: global efficiency) of the time varying negative connection EEG-fMRI graphs for beta frequency band (over 237 time windows; x is the index of time windows). Graph measures appear to dynamically change over time.

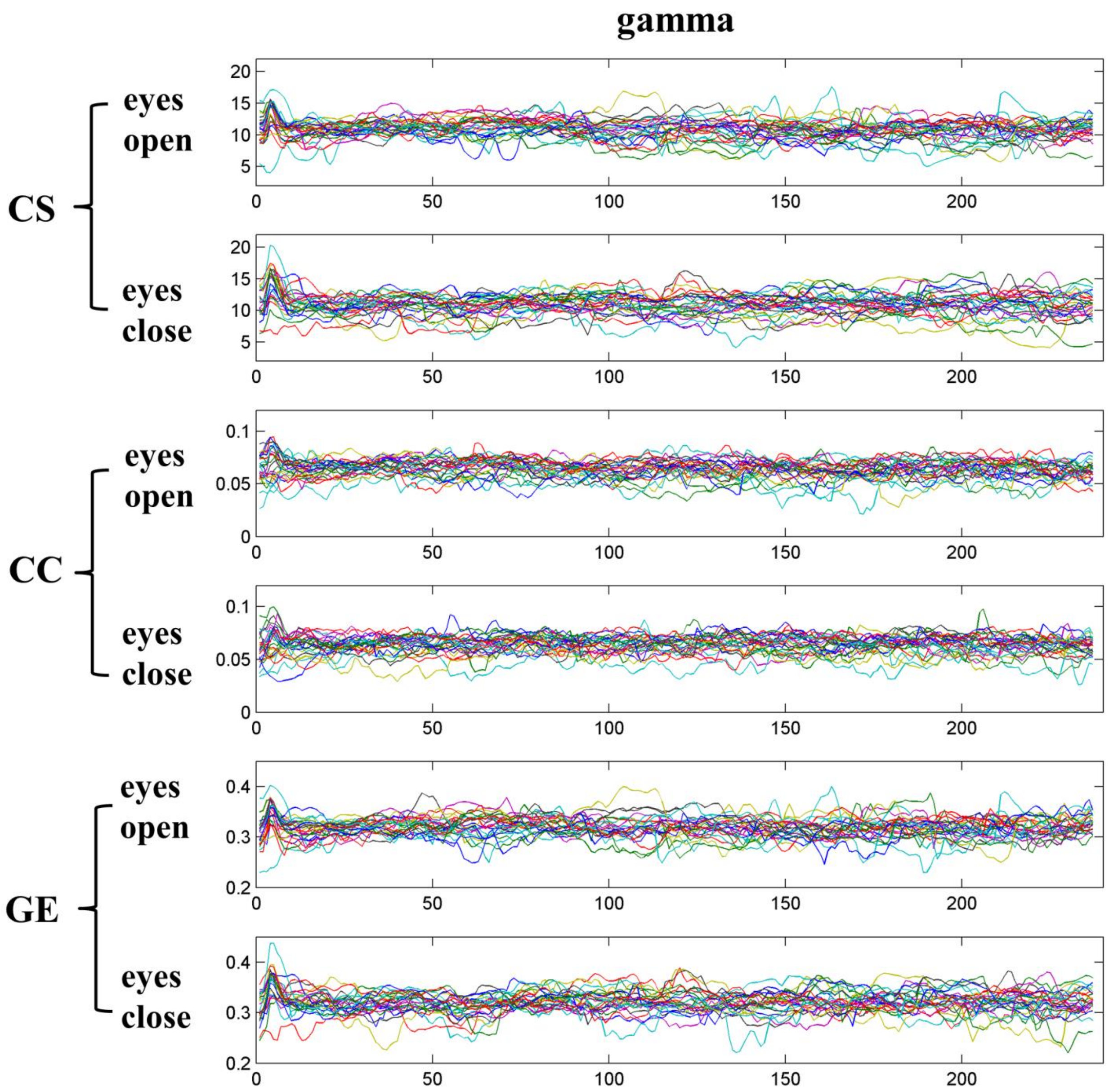

Figure S16. Global level graph metrics (CS: connectivity strength; CC: clustering coefficient; GE: global efficiency) of the time varying negative connection EEG-fMRI graphs for low gamma frequency band (over 237 time windows; x is the index of time windows). Graph measures appear to dynamically change over time.

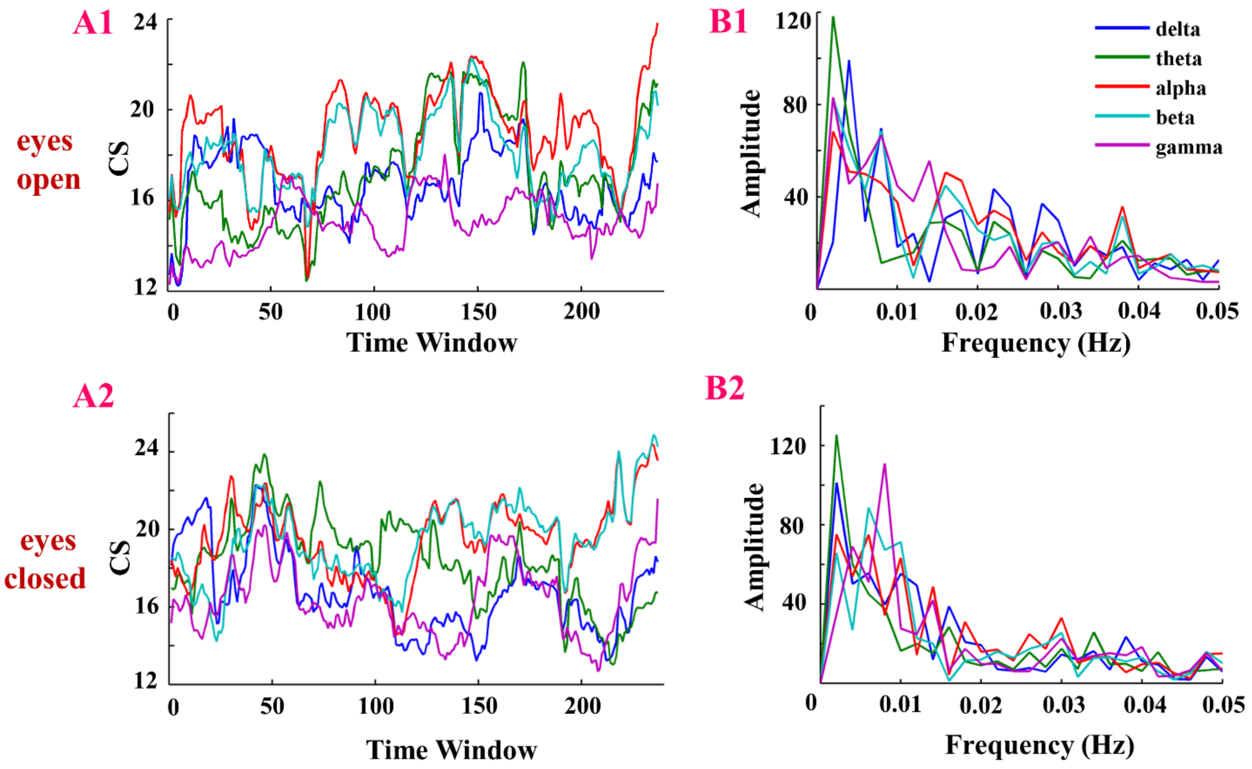

Figure S17. Dynamic global level connectivity strength for the time-varying positive connection graphs of a typical subject. (A1-A2) Time series of global level connectivity strength (CS) in all five frequency bands during eyes open and eyes closed. (B1-B2) Spectra of the time series of five bands in EO and EC.

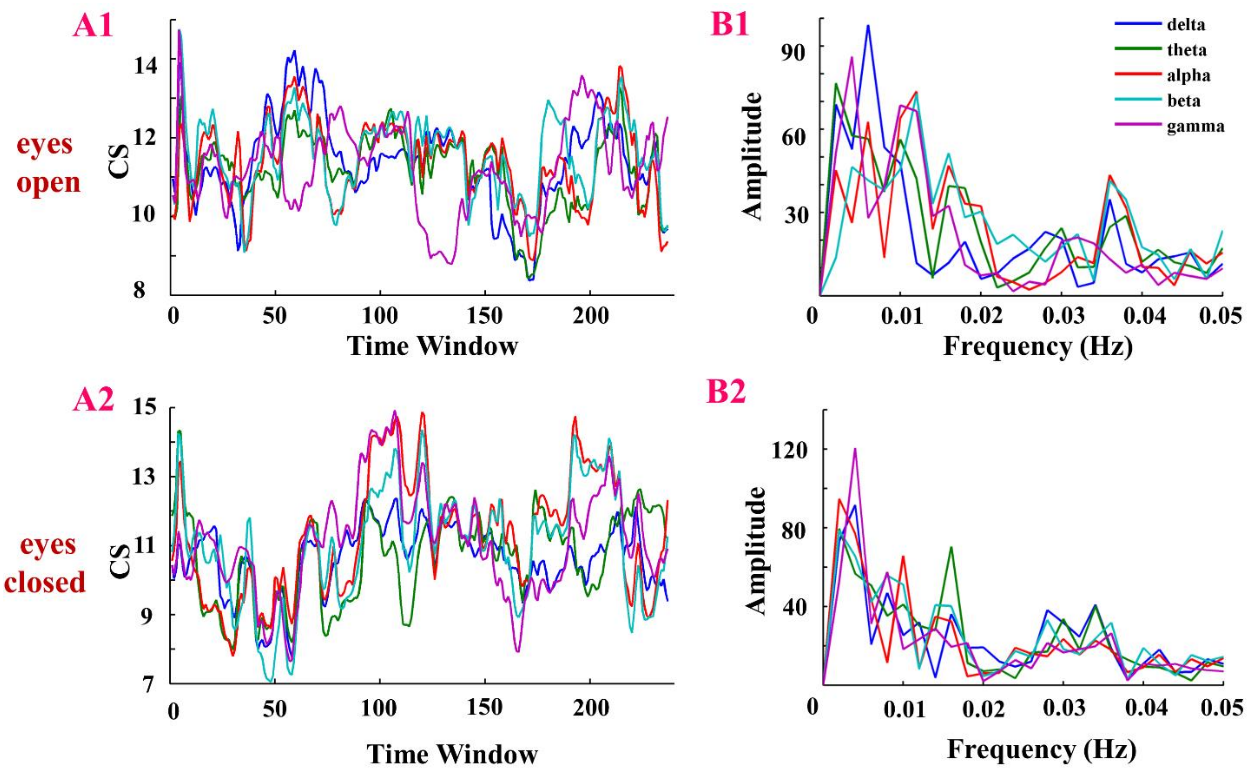

Figure S18. Dynamic global level connectivity strength for the time-varying negative connection graphs of a typical subject. (A1-A2) Time series of global level connectivity strength (CS) in all five frequency bands during eyes open and eyes closed. (B1-B2) Spectra of the time series of five bands in EO and EC.

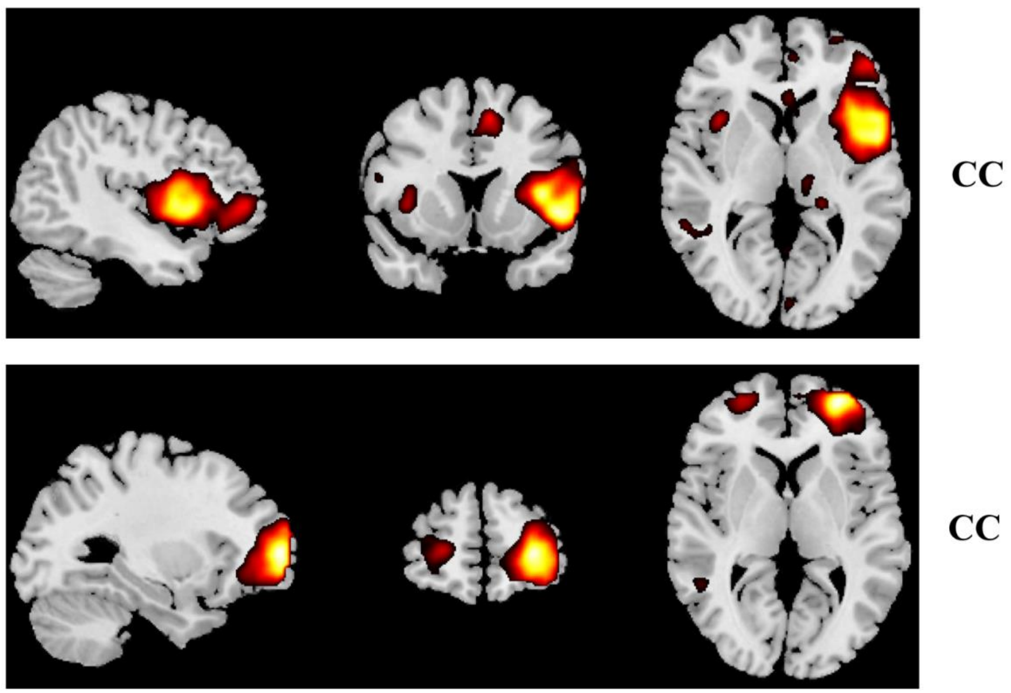

Figure S19. Two fMRI brain components which show higher VAR (variance) and LFA (amplitude of low frequency) of time-varying CS, CC and GE in eyes closed than in eyes open in the dynamic analysis for both positive and negative connection graphs. Both belongs to cognitive control (CC) components.

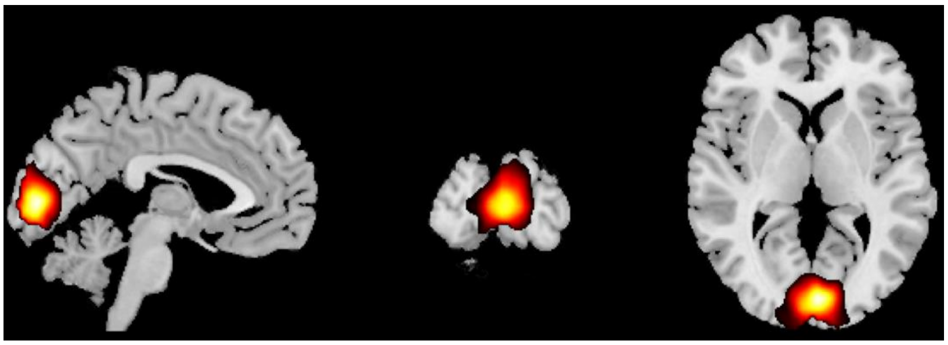

Figure S20. Spatial map of the visual component of which the main effect of eyes condition is significant on variance (VAR) and amplitude of low frequency (LFA) of connectivity strength (CS) and global efficiency (GE) in dynamic positive connection graph analysis.

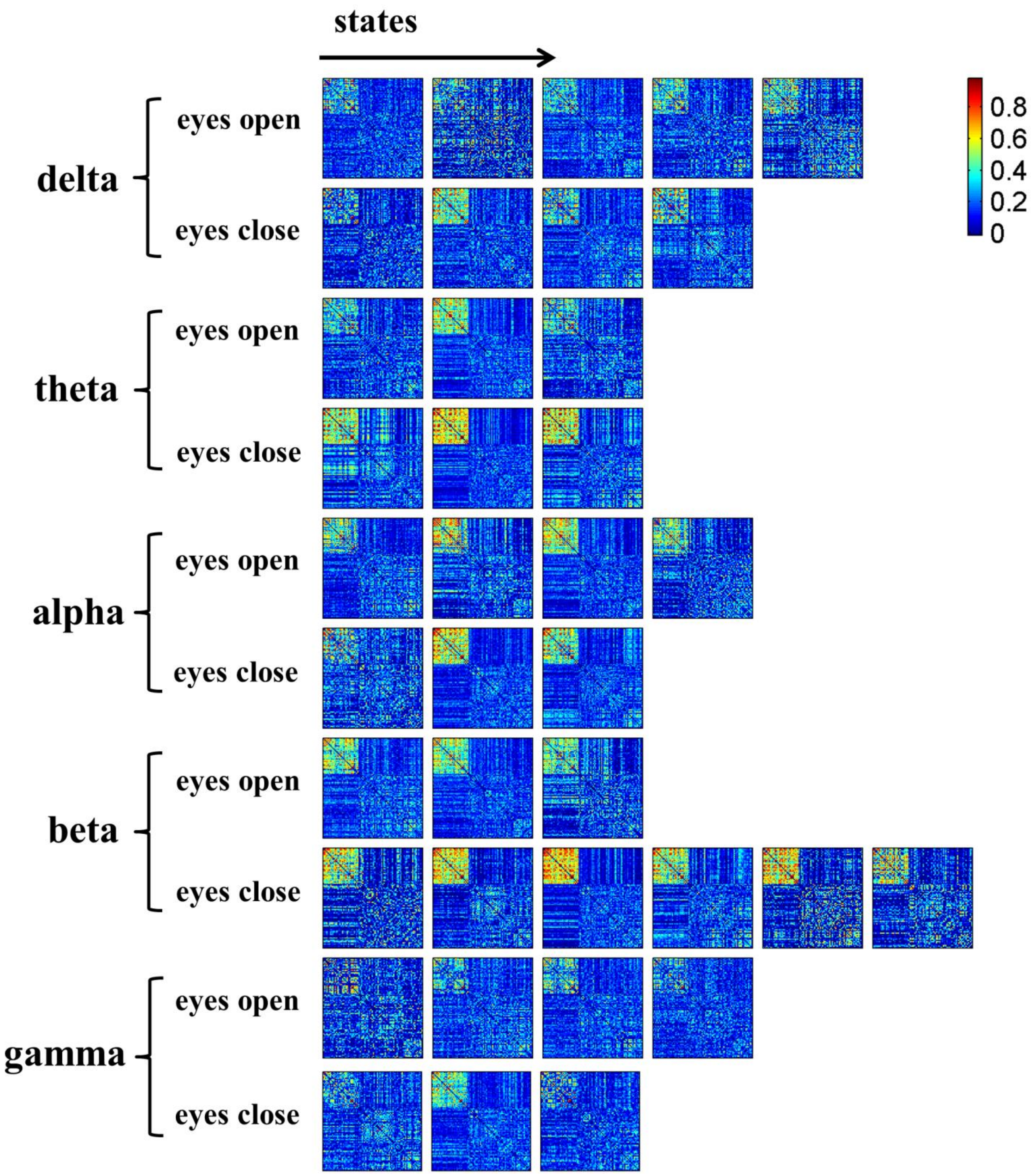

Figure S21. Structure of connectivity states for dynamic positive connection graphs in an example subject.

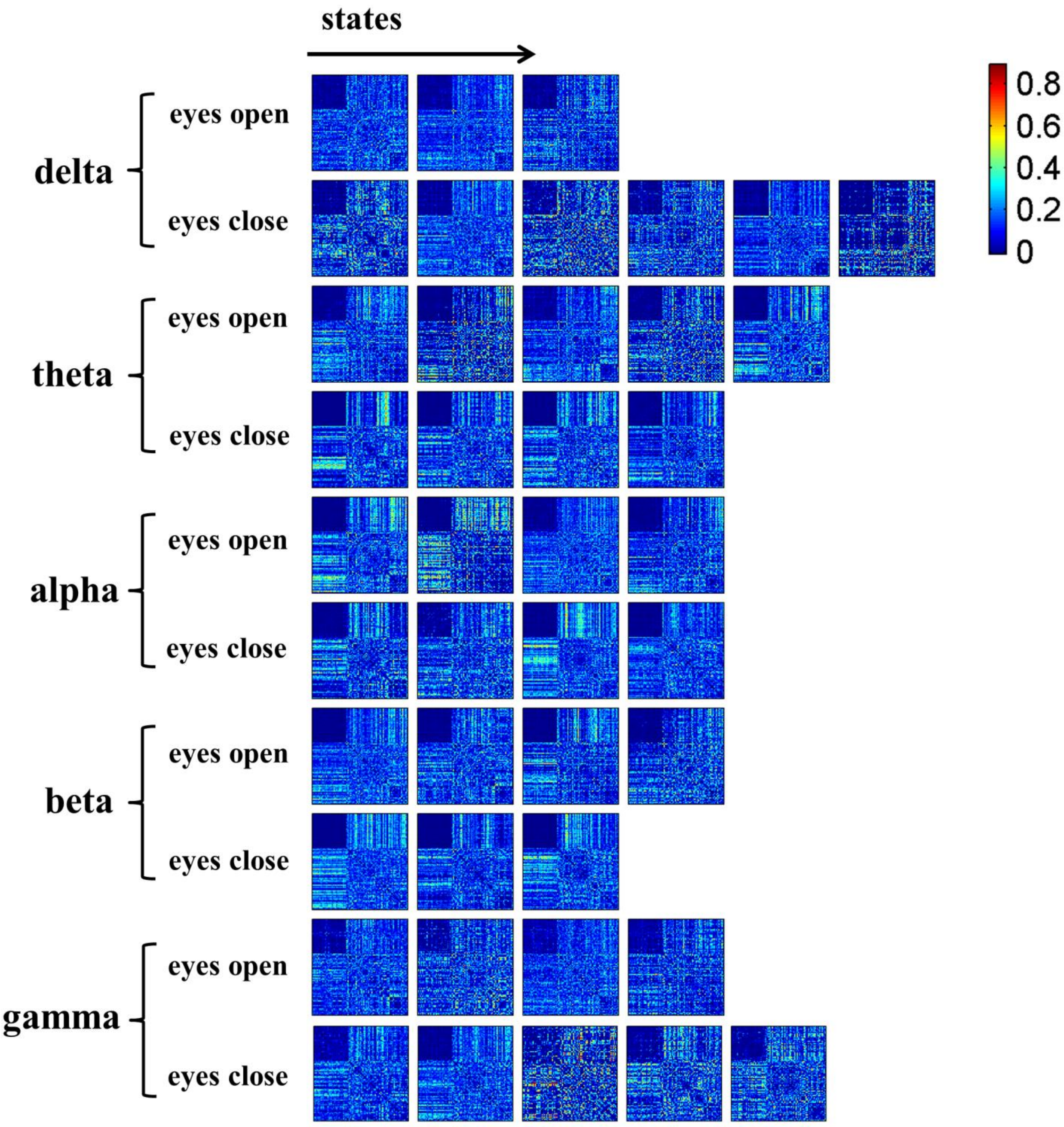

Figure S22. Structure of connectivity states for dynamic negative connection graphs in an example subject.

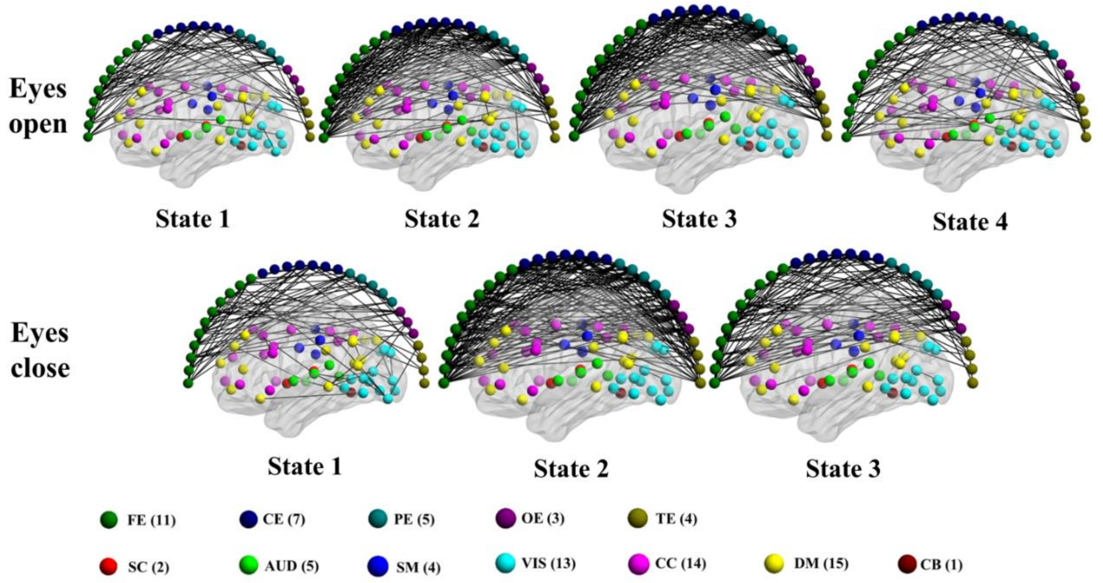

Figure S23. A visual view of connectivity states for positive connection graphs in alpha band of an example subject. Color dots inside the brain map indicate fMRI brain components. Color dots outside the brain map indicate EEG electrodes. (FE: frontal electrodes; CE: central electrodes; PE: parietal electrodes; OE: occipital electrodes; TE: temporal electrodes)

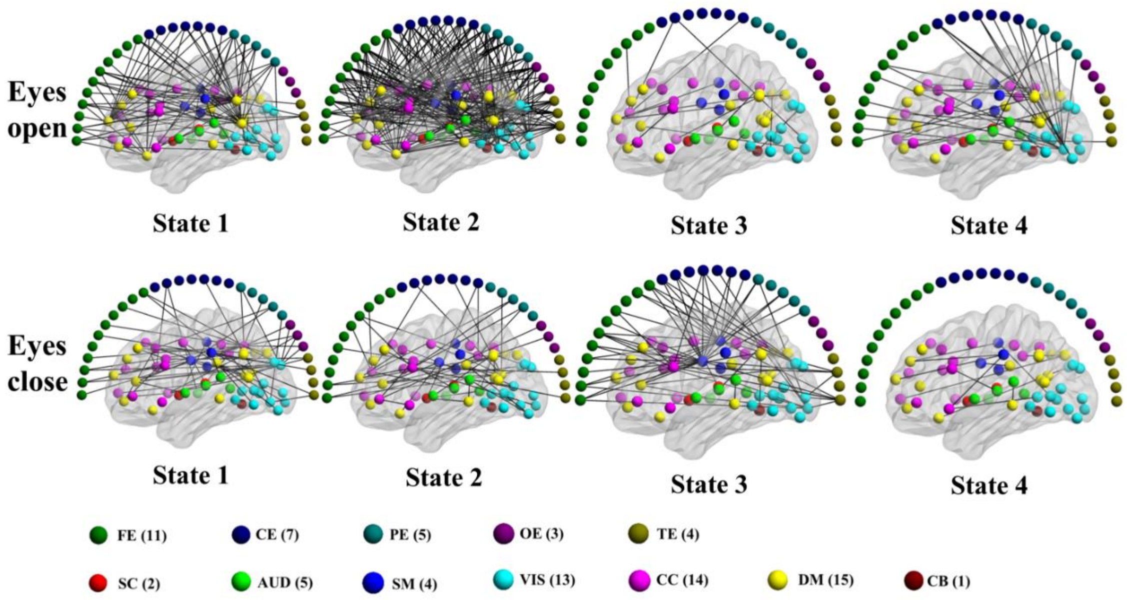

Figure S24. A visual view of connectivity states for negative connection graphs in alpha band of an example subject. Color dots inside the brain map indicate fMRI brain components. Color dots outside the brain map indicate EEG electrodes. (FE: frontal electrodes; CE: central electrodes; PE: parietal electrodes; OE: occipital electrodes; TE: temporal electrodes)
